# Enhancing Bioaccessibility of Plant Protein Using Probiotics: An In Vitro Study

**DOI:** 10.3390/nu15183905

**Published:** 2023-09-07

**Authors:** Maija Marttinen, Mehreen Anjum, Markku T. Saarinen, Ilmari Ahonen, Markus J. Lehtinen, Päivi Nurminen, Arja Laitila

**Affiliations:** 1 IFF Health and Biosciences, Danisco Sweeteners Oy, Sokeritehtaantie 20, 02460 Kantvik, Finland; 2 Vincit Plc, Helsinginkatu 15, 20500 Turku, Finland

**Keywords:** probiotics, plant protein, bioaccessibility, protein digestion, amino acids

## Abstract

As plant-based diets become more popular, there is an interest in developing innovations to improve the bioaccessibility of plant protein. In this study, seven probiotic strains (*Bifidobacterium animalis* subsp. *lactis* B420, *B. lactis* Bl-04, *Lactobacillus acidophilus* NCFM, *Lacticaseibacillus rhamnosus* HN001, *Lacticaseibacillus paracasei* subsp. *paracasei* Lpc-37, *Lactiplantibacillus plantarum* Lp-115, and *Lactococcus lactis* subsp. *lactis* Ll-23) were evaluated for their capacity to hydrolyze soy and pea protein ingredients in an in vitro digestion model of the upper gastrointestinal tract (UGIT). Compared to the control digestion of protein without a probiotic, all the studied strains were able to increase the digestion of soy or pea protein, as evidenced by an increase in free α-amino nitrogen (FAN) and/or free amino acid concentration. The increase in FAN varied between 13 and 33% depending on the protein substrate and probiotic strain. The survival of probiotic bacteria after exposure to digestive fluids was strain-dependent and may have affected the strain’s capacity to function and aid in protein digestion in the gastrointestinal environment. Overall, our results from the standardized in vitro digestion model provide an approach to explore probiotics for improved plant protein digestion and bioaccessibility of amino acids; however, human clinical research is needed to evaluate the efficacy of probiotics on amino acid absorption and bioavailability in vivo.

## 1. Introduction

The popularity of plant-based diets and plant-based meat and milk alternatives has grown over the past decade [1]. As the reliance on plant-based proteins as meaningful contributors to daily dietary protein intake increases, approaches to increase the bioaccessibilty of this essential nutrient may help individuals better meet their daily needs. Lactic acid bacteria (LAB) belonging to *Lactobacillus*, *Lactococcus*, and related genera, as well as *Bifidobacterium* spp., have been shown to express peptidases and proteolytic enzymes, and influence proteolysis in various food matrices [2]. The proteolytic properties of LAB have been utilized in the production of fermented foods for centuries. Several studies have confirmed that the fermentation of plant matrices with LAB can enhance the digestibility and bioaccessibility (i.e., the amount of a compound released from the matrix and available for absorption) of plant protein [3,4,5,6]. The impact of probiotic LAB on protein bioaccessibility and the digestion of protein in the upper gastrointestinal (GI) tract has been less studied. 

Probiotics are defined as “live micro-organisms that, when consumed in adequate amounts, confer a health benefit on the host” [7]. Food-derived bacteria and probiotics may impact the proteolysis and digestion of dietary protein in the GI tract, where endogenous digestive fluids and enzymes play a major role. Probiotics may induce the activity and/or production of digestive enzymes, enhance the absorptive capacity by improving the condition of the intestinal epithelium, or participate in the digestion process by producing proteolytic enzymes [8,9]. Furthermore, some probiotics are capable of synthesizing amino acids, including essential amino acids, in the intestinal tract, which may also benefit the host [10,11]. 

Earlier research has demonstrated that probiotic *Bacillus coagulans* GBI-30, 6086 increased protein hydrolysis and improved the digestion of soy, pea, and rice protein in vitro under simulated digestive conditions [12,13]. In a placebo-controlled human clinical trial, consumption of *B. coagulans* GBI-30, 6086 for 2 weeks was found to significantly increase postprandial amino acids in blood when ingested in combination with milk protein [14]. In another randomized placebo-controlled clinical trial, pea protein consumption with two *L. paracasei* strains for two weeks significantly increased the postprandial concentration of essential amino acids (EAAs) and branched chain amino acids (BCAAs) in blood compared to pea protein ingested with the non-probiotic placebo [8]. Concentrations of circulating BCAAs (isoleucine, leucine, and valine) have been observed to be significantly lower in vegans and lacto-ovovegetarians compared to omnivores [15]. A recent clinical study demonstrated that ingestion of high-quality plant protein blends, similar to high-quality whey protein (calculated Protein Digestibility Corrected Amino Acid Score, PDCAAS, equal to 1.0), resulted in a significantly lower postprandial response in blood EAAs and leucine compared to the ingestion of whey protein despite the blends being matched to whey protein in their EAAs and leucine contents [16]. Thus, improving the bioaccessibility and bioavailability of plant protein sources through different strategies, including the use of probiotics, could be considered especially with consumers following restricted diets.

The aim of the present study was to investigate the impact of selected commercial probiotic strains on protein digestion and bioaccessibility under conditions simulating digestion in the human upper GI tract. The digestion and bioaccessibility of nutrients and other dietary compounds can be evaluated using in vitro gastrointestinal digestion models that are often applied for screening purposes before conducting expensive and time-consuming animal or human clinical trials [17,18]. The in vitro digestion of protein was performed according to a standardized method for a static digestion model [17,19]. The protocol developed by a consensus of international experts describes the simplest standard parameters required to simulate the digestive conditions in the healthy adult GI tract [17,19]. Soy, pea, and whey proteins were digested in the presence of *Bifidobacterium animalis* subsp. *lactis* B420 (B420), *B. animalis* subsp. *lactis* Bl-04 (Bl-04), *Lactobacillus acidophilus* NCFM (NCFM), *Lacticaseibacillus rhamnosus* HN001 (HN001), *Lacticaseibacillus paracasei* subsp. *paracasei* Lpc-37 (Lpc-37), *Lactiplantibacillus plantarum* Lp-115 (Lp-115), and *Lactococcus lactis* subsp. *lactis* Ll-23 (Ll-23). Soy and pea protein breakdown without a probiotic was used as a control. Whey protein was included in the digestion experiments for comparison. To evaluate protein hydrolysis, samples collected at the beginning and after digestion were analyzed for free α-amino nitrogen content (FAN). Furthermore, soluble protein, free amino acids, and biogenic amines were determined from the samples. Exploring the effects of probiotics on protein digestion could potentially provide a novel approach for improving protein nutrition.

## 2. Materials and Methods

### 2.1. Cultivation of Probiotic Bacteria

Seven probiotic strains produced by IFF Health & Biosciences (Danisco USA Inc., Madison, WI, USA), B420 (DSM 32073), Bl-04 (DSM 33525), NCFM (DSM 33840), HN001 (DSM 22876), Lpc-37 (DSM 32661), Lp-115 (DSM 22266), and Ll-23 (DSM 33830), were selected for this in vitro study. Commercial probiotics were selected based on their genomes and ability to synthesize peptidases. Both lactobacilli (and related genera) and bifidobacterial strains were chosen. Fresh bacterial cultures were prepared from overnight cultures in either de Man, Rogosa, and Sharpe (MRS, Lab094; LAB M Ltd., Lancashire, United Kingdom) or modified *Bifidobacterium* medium 58 [20] and grown anaerobically at 37 °C as indicated in Table 1. Bacteria were grown until late-logarithmic stage with known target optical densities, and harvested through centrifugation (10 min, 4000× *g*, 4 °C). The bacteria-containing pellet was washed twice and resuspended in sterile saline solution. Optical density (OD600) was measured, and bacterial number was counted using OD600/cell count curve that was determined for each strain using flow cytometry in advance. 

### 2.2. Protein Ingredients

Protein ingredients used in protein digestion experiments were soy protein (SUPRO^®^; IFF, St. Louis, MO, USA), pea protein (TRUPRO™; IFF, St. Louis, MO, USA), and whey protein (BiPro, Davisco Foods International, Eden Prairie, MN, USA). Protein content of protein ingredient batches was determined through combustion method and was performed by NP Analytical Laboratories (St. Louis, MO, USA). Protein content of soy protein was 90.0%, pea protein 83.0%, and whey protein 92.0%, dry weight basis. Gamma-irradiation of 12 kGy, using a cobalt-60 γ source, was performed by Scandinavian Clinicals (Ionisos Baltics, Harjumaa, Estonia) to inactivate indigenous microbes present in the protein ingredients. The protein ingredients were kept in airtight jars at room temperature until use. 

### 2.3. Protein Digestion in Simulated Human Upper GI Tract 

Briefly, the static digestion process was simulated in three stages: oral, gastric, and small intestinal phase. The buffers and solutions for simulation, simulated saliva fluid (SSF), simulated gastric fluid (SGF), and simulated intestinal fluid (SIF), were prepared according to the INFOGEST protocol [17]. HCl and NaOH were used to adjust the pH and all the solutions were pre-warmed at 37 °C before use. Protein ingredient (2 g) was suspended in water (8 mL) and mixed with 10 mL of SSF containing amylase (10 mg/mL) (A3176, Sigma-Aldrich, Taufkirchen, Germany). Probiotic bacteria were grown as described above and inoculated at a total of 10^8^ bacteria in the oral phase. The pH of salivary fluid was adjusted to 6.9 ± 0.2, and the bottles were placed on a magnetic stirrer (200 rpm) in a water bath for 2 min at 37 °C. Then, 20 mL of SGF was added to the bottles with pepsin enzyme (4.5 mg/mL) (P7012, Sigma-Aldrich), and the pH was adjusted to 2.8 ± 0.2 and incubated for 2 h at 37 °C in a water bath with continuous magnetic stirring. Following the gastric stage, 40 mL of SIF was added to the flasks followed by pancreatin (16 mg/mL) (P3292, Sigma-Aldrich) and bile solution (30 mg/mL) (B8631, Sigma-Aldrich). The pH was adjusted to 6.5 ± 0.2 and the incubation was continued for 2 h at 37 °C in a water bath with mixing. 

Sampling was performed before starting the incubation for the oral phase (baseline) and after the digestion from the small intestinal phase. A sample from total digesta was collected for microbiological analyses. Soluble fraction was collected by spinning the samples at 10,000× *g* at 4 °C for 30 min. After centrifugation, the pellet was discarded, and supernatant was carefully separated, aliquoted, and immediately frozen at −80 °C. All samples were carefully kept on ice throughout sample collection and preparation to inactivate proteolytic activity after sampling. All protein digestions with or without probiotic strains were performed at least in three replicates.

### 2.4. Analysis of Soluble Protein

The bicinchoninic acid (BCA) assay was used for total protein quantitation through the microplate method, according to the manufacturer’s protocol (Thermo Scientific™ Pierce™ BCA protein assay, Rockford, IL, USA). Absorbance was measured at 562 nm using EnSight multimode plate reader (PerkinElmer, Turku, Finland). Bovine serum albumin, provided with the kit, was used as the protein standard. Whey protein samples were diluted 1000-fold while the soy and pea protein samples were diluted 100-fold in deionized water before analysis. Each dilution was analyzed in triplicate. 

### 2.5. Analysis of Free Amino Nitrogen and Degree of Hydrolysis

Free α-amino nitrogen (FAN) was measured to evaluate the extent of proteolysis in a sample using the o-phthaldialdehyde (OPA) method. The OPA method is frequently used to evaluate protein hydrolysis. In the method, nitrogen of terminal α-amino groups in free amino acids and small peptides released during hydrolysis react with the OPA reagent, producing a chromogenic complex. FAN was quantified using a manual assay procedure (K-PANOPA kit, Megazyme, Wicklow, Ireland) using isoleucine as a standard, according to manufacturer’s standard protocol. Briefly, the samples were diluted 10-fold in deionized water prior to analysis and were added to *N*-acetyl-L-cysteine solution. Absorbance was measured at 340 nm after a 2 min incubation at room temperature. OPA reagent was added, and absorbance was re-measured after a 15 min incubation. Measurements were performed in duplicate for each sample. Degree of hydrolysis (DH) was calculated from the FAN measurements using the following formula:

$$DH(\%)=\frac{FAN\left(d\right)}{FAN\left(tot\right)}*100(\%)$$

where FAN(d) is the concentration of released FAN after digestion and FAN(tot) is the total α-amino nitrogen measured after total acid hydrolysis (6 N HCl at 110 °C for 24 h) of the protein. 

### 2.6. Analysis of Free Amino Acids 

Free primary amino acids were determined using an automated pre-column derivatization procedure with OPA and reversed-phase high-performance liquid chromatography (HPLC), as described by Greene et al. [21] with modifications. In short, the samples were diluted 15-fold in deionized water and 100 µL of the diluted sample solution was mixed with 100 µL of the internal standard solution (350 µmol/L of L-norvaline in 0.1% trifluoroacetic acid) and incubated at 4 °C for 2 h to precipitate protein. After centrifugation, an aliquot of the supernatant (140 µL) was transferred into an Ultrafree-mc 10,000 NMWL microcentrifuge filter unit (Merck KGaA, Darmstadt, Germany) and centrifuged at 10,000× *g* for 1 h. The filtrate was used for the analysis. An Agilent 1260 Infinity II (Agilent, Waldbronn, Germany) chromatography system consisting of a quaternary pump, a column oven, a programmable injector, and a diode array detector was used for derivatization, separation, and detection of amino acids. One microliter of the sample solution was derivatized in the injector needle with a mixture of OPA and 3-mercapto-propionic acid reagent (10 mg/mL each, Agilent 5061-3335) in 0.4 M borate buffer pH 10.2 (Agilent 5061-3339) as previously described [21]. The separation of OPA-amino acid derivatives was performed on a Zorbax Eclipse Plus C18 column (2.1 mm × 100 mm, particle size 3.5 µm, Agilent) at 40 °C. A buffer solution consisting of 10 mM sodium phosphate–10 mM sodium borate at pH 8.2 was used as mobile phase A and a mixture of acetonitrile, methanol, and water (45:45:10) as mobile phase B. A gradient elution of A and B at flow rate of 0.42 mL/min was employed for the separation: 0–0.2 min, A = 98% and B = 2%; 0.2–7.7 min, a linear decrease in A to 43% and a linear increase in B to 57%; 7.8–8.3 min, A = 0% and B = 100%; 8.4–9 min, A = 98% and B = 2%. The OPA derivatives were detected at 338 nm and internal standardization method was used for the quantitation. 

### 2.7. Analysis of Biogenic Amines

Biogenic amines (2-methylbutylamine, 2-phenylethylamine, cadaverine, ethylamine, histamine, methylamine, putrescine, spermidine, spermine, tryptamine, and tyramine) were determined through HPLC as described by Saarinen [22] with modifications. In short, 100 µL of the sample solution was mixed with 100 µL of the internal standard (450 µmol/L of heptylamine in 0.4 M perchloric acid) and 200 µL of 0.4 M perchloric acid. After centrifugation, derivatization of biogenic amines in the supernatant was conducted as previously described [22]. The dansyl derivatives of biogenic amines were analyzed using an Agilent 1260 Infinity II chromatography system. The separation was performed under reversed-phase conditions using a Zorbax Eclipse Plus C18 column (2.1 mm × 100 mm, particle size 3.5 µm) at 55 °C and using a gradient elution with A: 0.02 M ammonium acetate buffer pH 5 in water (75%)–acetonitrile (25%), and B: 0.02 M ammonium acetate buffer pH 5 in water (15%)–acetonitrile (85%). The following gradient of A and B was employed for the separation: initial A = 100% and B = 0%; 0–1 min, a linear decrease in A to 75% and a linear increase in B to 25%; 1–4 min, A = 75 and B = 25%; 4–16 min, a linear decrease in A to 0% and a linear increase in B to 100%; 16–20 min, A = 0% and B = 100%; 20.1–25 min, A = 100% and B = 0%. The flow rate of the mobile phase was 0.3 mL/min and the dansyl derivatives of biogenic amines were detected at 254 nm and quantitated using the internal standardization method.

### 2.8. Microbiological Analyses

The plate counting method on MRS agar was used to enumerate bacteria at the beginning and after in vitro digestion. Plates were incubated anoxically at 37 °C in Mitsubishi Anaeropack jar (Thermo Scientific, Rockford, IL, USA) with two Anaerogen sachets (Oxoid, Thermo Scientific, Wesel, Germany). Colonies were counted daily under aerobic conditions for three consecutive days. The probiotic survival is reported as log10 CFU. 

### 2.9. Statistical Analysis 

After testing for normality of the data, two-way ANOVA was used to analyze FAN, soluble protein, and biogenic amines in the in vitro digestions using GraphPad Prism (version 8.3.1, GraphPad Software, LLC, LaJolla, CA, USA). For post hoc analyses, Šidak’s multiple comparisons test was performed to compare differences between timepoints (baseline vs. after digestion), and Dunnett’s multiple comparisons test was applied to compare differences between probiotic and control treatments within timepoint. Statistical significance was set at *p* < 0.05.

Free amino acids were analyzed from soy and pea protein samples collected at the beginning and after protein digestion. For statistical analysis, when concentration of detected compound was below the quantitation limit, the value was adjusted the same as the quantitation limit. The treatment groups were compared in the absolute change in amino acid content from baseline to the levels measured after digestion. The data were log-transformed and analyzed using a robust linear model using Huber’s M-estimator that is not adversely affected by sporadic outliers in the data. A total of two models were fitted, one for soy and another for pea data. *p*-values were corrected for false discovery rate using Benjamini-Hochberg method. This analysis was performed using R version 4.1.0 (18 May 2021) [23], with packages MASS [24] for the robust regression and tidyverse [25] for data processing and figures. 

## 3. Results

### 3.1. Effect of Probiotics on Protein Solubility 

**Figure 1:**
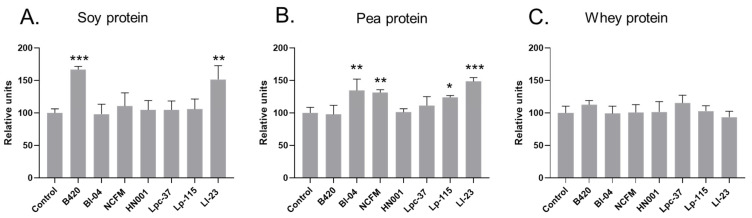
Content of soluble protein relative to control digestion (without a probiotic) measured after in vitro digestion of soy (**A**), pea (**B**), and whey (**C**) protein. B420 = *Bifidobacterium animalis* subsp. *lactis* B420; Bl-04 = B. lactis Bl-04; NCFM = *Lactobacillus acidophilus* NCFM; HN001 = *Lacticaseibacillus rhamnosus* HN001; Lpc-37 = *Lacticaseibacillus paracasei* subsp. *paracasei* Lpc-37; Lp-115 = *Lactiplantibacillus plantarum* Lp-115; and Ll-23 = *Lactococcus lactis* subsp. *lactis* Ll-23. Statistical difference between probiotic treatment and digestion without added probiotic (control), * *p* < 0.05, ** *p* < 0.01, *** *p* < 0.001. In the figure, results without an asterisk are non-significant (*p* ≥ 0.05).

The solubility of soy protein did not change from baseline after digestion without a probiotic (Supplementary Table S1); however, a significant increase in soluble soy protein content was observed after digestion with B420 and Ll-23 compared to the non-probiotic control digestion (Figure 1, Supplementary Table S1). The content of soluble soy protein was 67% higher in B420 digests (*p* < 0.001) and 52% higher in Ll-23 digests (*p* = 0.001) compared to soy protein control digests. 

The solubility of pea protein was significantly increased in all pea protein digestion samples from baseline values (Supplementary Table S1). Compared to the control digests, the content of soluble pea protein was significantly higher after digestion with Bl-04 (+35%; *p* = 0.003), NCFM (+31%; *p* = 0.008), Lp-115 (+24%; *p* = 0.0495), and Ll-23 (+48%; *p* < 0.001) (Figure 1). When comparing the absolute changes in soluble protein, the change from baseline remained significant with Bl-04 (*p* = 0.045) and Ll-23 (*p* = 0.0013) and close to significant with NCFM (*p* = 0.053). A higher average change from baseline was also observed with Lp-115, but it was not significant (*p* = 0.092) (Supplementary Table S1). The solubility of whey protein did not change from baseline after digestion in the presence or absence of a probiotic and there was no difference in the soluble protein content between control and probiotic whey protein digests. 

### 3.2. Effect of Probiotics on Protein Hydrolysis and Release of Free Amino Nitrogen

To evaluate protein hydrolysis after the intestinal phase of the upper GI tract simulation, the concentration of free FAN was measured at the beginning and after protein digestion. Additionally, DH was calculated for the intestinal digesta samples. The FAN concentrations measured at baseline did not differ between the control and probiotic simulations within the soy, pea, and whey protein samples. The concentration of FAN increased significantly from baseline in all digestion samples, confirming increased protein hydrolysis in the simulation model (*p* < 0.001, for all; Table 2). The concentration on FAN was at a higher level in whey protein digests than in soy and pea protein digests.

Compared to the control digestion of soy protein, higher FAN concentrations were observed in soy protein digests with Bl-04 (*p* < 0.001; mean increase relative to control +33%), NCFM (*p* = 0.0001; mean increase relative to control +31%), HN001 (*p* < 0.001; mean increase relative to control +31%), Lp-115 (*p* = 0.006; mean increase relative to control +23%), Lpc-37 (*p* = 0.010; mean increase relative to control +20%), and B420 (*p* = 0.027; mean increase relative to control +19%) (Table 2). In addition, a significantly higher DH was seen in intestinal digests of Bl-04, NCFM, and HN001 compared to the control (Table 2). A higher average in DH value was also detected in Lp-115 digests in comparison to the soy protein control digest, but the difference was non-significant (*p* = 0.096).

In pea protein digests, the concentration of FAN was significantly higher with NCFM (*p* = 0.005; mean increase relative to control +16%) and B420 (*p* = 0.031; mean increase relative to control +13%) when compared to the control (Table 2). Digestion of pea protein with NCFM resulted in a higher average in DH compared to control digestion, and the difference was close to significant (*p* = 0.068).

Digestion of whey protein in the presence of Lpc-37 resulted in a significantly higher FAN concentration compared to the control (*p* = 0.019; mean increase relative to control +20%). The baseline FAN values of whey protein showed more variation, and when comparing the absolute changes in intestinal digesta samples of control and probiotic treatments, there were no differences in whey protein digests between the control and probiotic treatments (Table 2). Furthermore, the DH of whey protein was not impacted by the tested probiotics.

### 3.3. Effect of Probiotics on Release of Free Amino Acids from Soy and Pea Protein

**Figure 2:**
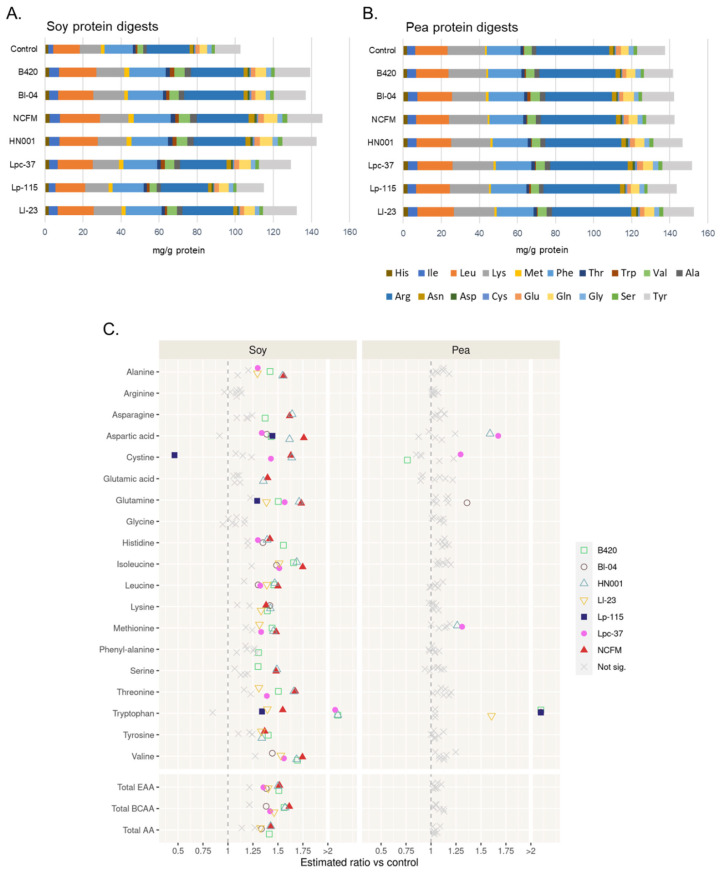
Free amino acids in the soluble phase after in vitro digestion of soy and pea protein. (**A**) Concentration of free amino acids (mean values, mg/g protein in a simulation) in soy protein digests in the absence (control treatment) or presence of the tested probiotic strains. (**B**) Concentration of free amino acids (mean values, mg/g protein in a simulation) in pea protein digests in the absence (control treatment) or presence of the tested probiotic strains. In (**A**,**B**), essential amino acids (His-Val) are listed first from left to right in the bar graphs, and then non-essential amino acids (Ala-Tyr). (**C**) Comparison of probiotic digests to control digests in terms of absolute change in free amino acid content from baseline to the levels measured after digestion. The comparisons are reported as ratios against the control treatment where a ratio of 1.0 denotes no difference; ratio < 1, lesser change than in control; and ratio > 1, greater change than in control. B420 = *Bifidobacterium animalis* subsp. *lactis* B420; Bl-04 = *B. lactis* Bl-04; NCFM = *Lactobacillus acidophilus* NCFM; HN001 = *Lacticaseibacillus rhamnosus* HN001; Lpc-37 = *Lacticaseibacillus paracasei* subsp. *paracasei* Lpc-37; Lp-115 *= Lactiplantibacillus plantarum* Lp-115; and Ll-23 = *Lactococcus lactis* subsp. *lactis* Ll-23; total EAA = total essential amino acids (His, histidine; Ile, isoleucine; Leu, leucine; Lys, lysine; Met, methionine; Phe, phenylalanine; Thr, threonine; Trp, tryptophan; Val, valine); total BCAA = total branched chain amino acids (isoleucine, leucine, valine); total AA = total amino acids.

To further evaluate the impact of probiotics on increased soy and pea protein hydrolysis, the release of free amino acids was analyzed from soy and pea protein digests. For comparison, free amino acids were measured from the whey control digests without a probiotic. Concentrations of total free amino acids, EAAs, and BCAAs were significantly increased after protein digestion in all samples (Supplementary Table S2). The increase from baseline in the concentration of total free amino acids was approximately 7-, 14-, and 13-fold in soy, pea, and whey protein samples, respectively, after the simulated digestion. Digestion of whey protein without a probiotic resulted in a higher concentration of released total amino acids compared to digestion of soy and pea protein (Supplementary Figure S1). In soy and pea protein control digesta samples, the most abundant free EAAs were leucine (13% and 12% of all free amino acids, respectively), lysine (11% and 14%, respectively), and phenylalanine (15% and 13%, respectively) and the most abundant free non-essential amino acids was arginine (22% and 28%, respectively), followed by tyrosine (13% and 11%, respectively) (Figure 2A,B and Figure S1).

Digestion of soy protein resulted in a significantly higher concentration of total free amino acids in the presence of NCFM (+43%, *p* < 0.01), HN001 (+43%, *p* < 0.01), B420 (+42%, *p* < 0.01), Bl-04 (+34%, *p* < 0.05), and Ll-23 (+33%, *p* < 0.05) when compared with digestion without a probiotic (Figure 2C, Supplementary Table S3). In addition, all strains except Lp-115 significantly increased the concentration of total EAAs and BCAAs in soy protein digests compared to the control digest. Soy protein digests of NCFM demonstrated the greatest improvement in EAAs (+52%, *p* < 0.01) and BCAAs (+61%, *p* < 0.001) compared to the control digests, followed by HN001 (EAAs +51%, *p* < 0.01; BCAAs +57%, *p* < 0.001), B420 (EAAs +51%, *p* < 0.01; BCAAs +56%, *p* < 0.001), Ll-23 (EAAs +41%, *p* < 0.01; BCAAs +46%, *p* < 0.01), Lpc-37 (EAAs +36%, *p* < 0.05; BCAAs +42%, *p* < 0.01), and Bl-04 (EAAs +39%, *p* < 0.01; BCAAs +38%, *p* < 0.01). Additionally, digestion of soy protein in the presence of NCFM and HN001 resulted in the increase in the majority of individual amino acids compared to the control without a probiotic (Figure 2C, Supplementary Table S3). Compared to the control digestion, the concentration of methionine was significantly increased in soy protein digests with NCFM (+48%, *p* < 0.01), HN001 (+46%, *p* < 0.01), B420 (+44%, *p* < 0.01), Lpc-37 (+33%, *p* < 0.05), and Ll-23 (+32%, *p* < 0.05) when compared with the control. Furthermore, digestion of soy protein resulted in a significantly higher free lysine concentration with NCFM (+38%, *p* < 0.01), HN001 (+42%, *p* < 0.01), B420 (+40%, *p* < 0.01), Bl-04 (+42%, *p* < 0.01), and Ll-23 (+33%, *p* < 0.05) when compared to the control. 

Pea protein digestion with probiotics did not differ in the concentrations of total free amino acids, EAAs, and BCAAs from the control digestion without a probiotic (Figure 2C, Supplementary Table S4). However, concentrations of some individual amino acids (aspartic acid, glutamine, cystine, and methionine) were significantly higher in specific probiotic pea protein digests compared to the control. The concentration of the EAA methionine was significantly higher with Lpc-37 (+31%, *p* < 0.01) and with HN001 (+26%, *p* < 0.05) when compared with the control pea digests (Figure 2C, Supplementary Table S4). 

Although we report significant increases in free tryptophan, in soy protein digests of B420, HN001, NCFM, Lpc-37, Lp-115, and Ll-23 and in pea protein digests of B420, Bl-04, Lp-115, and Ll-23, the results should be considered only indicative of the increased release of tryptophan due to frequent observations of HPLC peaks below quantitation limits, especially in the control soy and pea protein digests. 

Overall, digestion of soy protein with the studied probiotics resulted in more observed differences in released amino acids between the control digest and probiotic digests than for pea protein digestion. 

### 3.4. Effect of Probiotics on Production of Biogenic Amines during Digestion of Soy and Pea Protein

**Figure 3:**
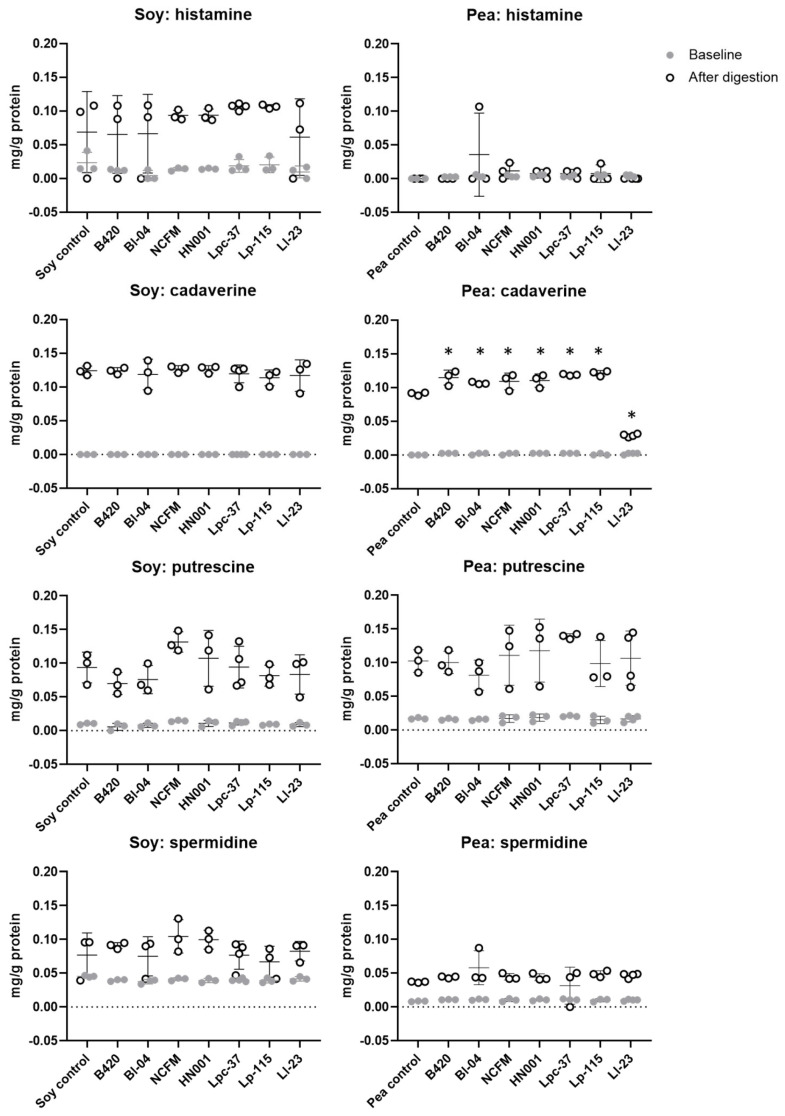
Concentration of cadaverine, histamine, putrescine, and spermidine before and after in vitro digestion of soy and pea protein. Other analyzed biogenic amines (tyramine, 2-methylbutylamine, 2-phenylethylamine, ethylamine, methylamine, spermine, tryptamine) were not detected in samples collected at baseline or after digestion. Results are expressed as log10 CFU (colony-forming units) presented as the mean with standard deviation. B420 = *Bifidobacterium animalis* subsp. *lactis* B420; Bl-04 = *B. lactis* Bl-04; NCFM = *Lactobacillus acidophilus* NCFM; HN001 = *Lacticaseibacillus rhamnosus* HN001; Lpc-37 = *Lacticaseibacillus paracasei* subsp. *paracasei* Lpc-37; Lp-115 *= Lactiplantibacillus plantarum* Lp-115; and Ll-23 = *Lactococcus lactis* subsp. *lactis* Ll-23. * Statistical difference between control and probiotic after digestion, *p* < 0.05.

Since specific probiotics were capable of increasing protein hydrolysis of soy and pea protein under the simulated digestive conditions, the concentration of biogenic amines was analyzed from all soy and pea protein digestion samples. For whey protein, biogenic amines were measured only from the whey control samples without a probiotic for comparison (Supplementary Figure S2). Of the analyzed biogenic amine compounds, only histamine, cadaverine, putrescine, and spermidine were detected (Figure 3). Tyramine was not detected in the digestion samples with or without probiotics. The concentration of histamine increased after digestion in all samples with no statistically significant difference between the tested probiotics and non-probiotic control. We noted increased levels of putrescine and spermidine after digestion regardless of probiotic addition, demonstrating that the digestion process as such contributes to the formation of biogenic amines. Concentrations of cadaverine and putrescine were increased after soy protein digestion with no statistically significant differences between the probiotics and control. In contrast, digestion of pea protein with probiotics resulted in significantly higher cadaverine levels compared to digestion without a probiotic, except with Ll-23 that significantly decreased the concentration of cadaverine. The increased level of cadaverine by probiotics in pea protein digests was, however, not different from the level found after the control digestion of soy protein.

### 3.5. Probiotic Survival after the Simulated Digestion 

**Figure 4:**
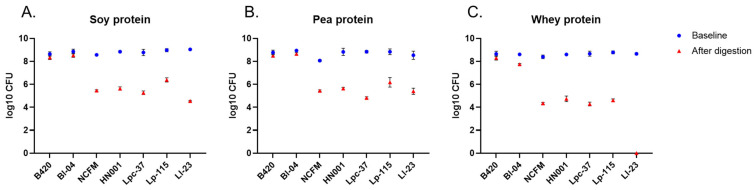
Total counts of probiotics at baseline and after in vitro digestion of soy (**A**), pea (**B**), and whey (**C**) protein. Results are expressed as log10 CFU (colony-forming units) presented as the mean with standard deviation. B420 = *Bifidobacterium animalis* subsp. *lactis* B420; Bl-04 = *B. lactis* Bl-04; NCFM = *Lactobacillus acidophilus* NCFM; HN001 = *Lacticaseibacillus rhamnosus* HN001; Lpc-37 = *Lacticaseibacillus paracasei* subsp. *paracasei* Lpc-37; Lp-115 *= Lactiplantibacillus plantarum* Lp-115; and Ll-23 = *Lactococcus lactis* subsp. *lactis* Ll-23.

The tested probiotics were added as live bacterial cultures to the oral stage at the beginning of the simulated digestion. Probiotic survival was evaluated by plate-counting simulation samples collected at the beginning and after the in vitro protein digestion. Bifidobacterial strains B420 and Bl-04 showed better survival under the digestive conditions compared to other tested strains as their counts were maintained close to the inoculated levels (~10^8^ CFU) (Figure 4). B420 counts were reduced by 0.3 log in all digests. Similarly, Bl-04 was reduced by 0.3 log in soy and pea protein digests and by 0.9 log in whey protein digests. The log reductions for B420 and Bl-04 were significantly smaller than for other strains in all protein digests (*p* < 0.001 for all comparisons between B420 or Bl-04 and other strains). The mean reduction in plate counts per digested protein is shown in Supplementary Table S5. In general, probiotic counts were at a higher level in soy and pea protein digests than in whey protein digests. For Ll-23, no viable cells were detected after digestion with whey protein (Figure 4C).

## 4. Discussion

In the present study, the impact of seven commercial probiotics on the digestion of selected protein substrates was evaluated under conditions mimicking the human UGIT. Our results demonstrate that specific probiotic strains were able to improve parameters related to the digestion and bioaccessibility of soy and pea protein under digestive conditions in vitro (Table 3). Of the tested probiotic strains, *B. lactis* B420, *B. lactis* Bl-04, *L. acidophilus* NCFM, *L. rhamnosus* HN001, *L. paracasei* Lpc-37, and *L. plantarum* Lp-115 significantly increased hydrolysis of soy protein compared to the control digestion without a probiotic. Compared to the control digestion of soy protein, the addition of a probiotic increased protein hydrolysis, measured as FAN, by up to 33%, with Bl-04, NCFM, and HN001 having the greatest efficacy. For pea protein, *L. acidophilus* NCFM and *B. lactis* B420 significantly increased FAN compared to the pea protein control digestion. Probiotics did not improve the hydrolysis or solubility of whey protein in the applied digestion model.

Protein solubility is one determinant affecting the digestibility of a protein source, with soluble protein being more easily accessed by digestive enzymes in the gastrointestinal tract [5]. In the present study, *B. lactis* B420 and *L. lactis* Ll-23 significantly increased the soluble protein content of the intestinal digests of soy protein and *B. lactis* Bl-04, *L. acidophilus* NCFM, *L. lactis* Ll-23, and *L. plantarum* Lp-115 in the pea protein digests (Figure 1). Interestingly, protein hydrolysis and protein solubility were not consistently increased in the protein digesta samples, through the same probiotic treatment (Figure 1 and Table 2). One explanation for this may be that the probiotic’s mode of action for improving these two outcomes are different. The detection method of FAN by OPA is based on the detection of terminal amino nitrogen in di- and tripeptides and free amino acids released after protein hydrolysis in the intestinal digesta samples [26]. Although the solubility of protein is increased by the degree of hydrolysis, it is also influenced by multiple factors including the surface characteristics and tertiary structure of the protein, and the surrounding pH [27,28]. Additionally, the solubility of dietary plant protein is greatly affected by anti-nutrient factors such as phytic acid and tannins [29]. Plant protein isolates and concentrates such as those included in our study have better digestibility than their native proteins due to the removal of these anti-nutrients during manufacturing processes [29]. It has been, however, demonstrated that the solubility and digestibility of plant protein can be increased by the enzymatic activity of bacteria, resulting in reduced levels of phytic acid and tannins [5,29]. We suggest that probiotics B420 and Ll-23 with soy protein, and Bl-04, NCFM, Ll-23, and Lp-115 with pea protein, could have improved the solubility of the respective proteins by impacting the tertiary or the secondary structures of the protein substrate and/or aided in the protein digestion resulting in the formation of larger peptides not reflected in the FAN value. 

Previous research has demonstrated that probiotic *B. coagulans* GBI-30, 6086 was able to improve the soy, pea, and rice digestion rate in vitro [12,13] and increase postprandial amino acids in blood when consumed in combination with milk protein [14]. In a randomized placebo-controlled clinical trial, pea protein consumption with two *Lacticaseibacillus paracasei* strains LP-DG (CNCM I-1572) and LPC-S01 (DSM 26760) for two weeks significantly increased the postprandial concentration of EAAs and BCAAs in blood compared to pea protein ingested with a non-probiotic placebo [8]. Before the clinical trial, a similar kind of in vitro screening under simulated digestive conditions was performed for the *L. paracasei* strains [8] as in our study. There are several potential mechanisms of probiotics to improve the bioaccessibility and bioavailability of amino acids in the upper GI tract. Probiotic bacteria have been shown to increase the production of digestive enzymes and to enhance their efficacy [9,13]. Probiotics may aid in protein digestion by producing proteolytic enzymes for the host’s benefit or act in synergy with digestive enzymes secreted by the host [9,12,13]. Furthermore, probiotics may enhance the absorptive capacity of the intestinal epithelium [30]. 

The probiotic bacteria themselves may utilize protein by breaking protein into smaller peptides and release free amino acids into their surroundings [31]. Peptidases produced by bacteria can be secreted into the extracellular environment or can be released in the gastrointestinal environment when bacteria lyse [9,12]. These actions are dependent on the proteolytic machinery of the probiotic and are species- and strain-dependent. In LAB, the proteolytic system comprises three components: cell-envelope-associated proteinase that initiates hydrolysis of extracellular protein, specific transport systems for peptides and amino acids, and various intracellular peptidases [31,32]. The genomes of LAB, including species of *Lactobacillus* and related genera, and *Lactococcus* species, in general, encode a high number and variety of proteases, peptidases, amino acid permeases, and transport systems [31,32]. In our study, *L. acidophilus* NCFM demonstrated good efficacy on protein hydrolysis with both soy and pea protein (Table 2). *L. acidophilus* NCFM is known for a large number of peptidases and proteases at the genomic level [33]. NCFM has genes for cell-envelope proteases (Prtp) and genes for Prtp maturation [33], suggesting that NCFM can digest large proteins extracellularly and generate small peptides and essential amino acids that are internalized by its oligopeptide transporters (Opp), di- and tripeptide transporter systems (Dpp), and amino acid permeases [32,33]. In addition, several endopeptidases that hydrolyze peptide bonds within an oligopeptide have been identified in NCFM [32]. Even though some of the probiotics tested in the present study demonstrated reduced cell counts in the small intestinal digests compared to baseline counts, the release of cytoplasmic peptidases upon bacteria lysis may explain the efficacy of probiotics with poorer survival. This would in part explain the improved protein hydrolysis seen with *L. acidophilus* NCFM.

In a genome-wide comparative analysis of different LAB, endopeptidases were not identified in *Lactococcus* species, and within *lactococci,* the presence of cell-envelope-associated proteinase was found to be species- and strain-dependent [32]. Less is known about proteolytic systems in bifidobacteria [2]. In *B. animalis* spp. *lactis* strain DSM 10140T, endopeptidase PepO was characterized but the *B. lactis* strain was not active in degrading extracellular protein (casein) [34]. 

Of the seven tested strains in our study, all except *L. plantarum* Lp-115 significantly increased the concentration of total EAAs and BCAAs in soy protein digests compared to control digests (Figure 2). Moreover, concentrations of many of the individual amino acids were significantly increased by *L. acidophilus* NCFM, *L. rhamnosus* HN001, *L. paracasei* Lpc-37, *B. lactis* B420, and *B. lactis* Bl-04 (Figure 2C). For pea protein, the changes between control digestion and probiotic treatments were more modest (Figure 2C). Interestingly, the concentration of free methionine was significantly increased both in soy and pea protein digests with *L. paracasei* Lpc-37 and with *L. rhamnosus* HN001 (Figure 2C). Increasing the release of the EAA methionine from the protein matrix may be one way to improve the nutritional value of protein sources such as soy and pea protein that are relatively low in methionine [35]. Soy protein also has a relatively low content of the EAA lysine. Digestion of soy protein in the presence of Bl-04, B420, HN001, NCFM, and Ll-23 resulted in a significantly higher free lysine concentration in the digests when compared with control digests. Bacteria in the small and large intestine are capable of synthesizing amino acids, including EAAs; however, the role of microbial amino acid synthesis on the host is not well established [10,11]. Whether the increase in free amino acids, such as methionine and lysine, in intestinal digests resulted from increased proteolysis or bacterial synthesis cannot be differentiated based on our study design. 

During proteolysis and bacterial enzyme activity, bioactive nitrogen containing compounds with diverse effects on the host are being produced. Biogenic amines are amino acid derivatives formed during bacterial metabolism but also synthesized endogenously in humans [36]. Biogenic amines have important biological functions; however, ingestion of food with high concentrations of biogenic amines can provoke toxicological reactions [36]. Histamine and tyramine are considered the most harmful biogenic amines, and a high intake of histamine from foods can cause food poisoning and adverse effects that are similar to symptoms in allergic reactions [37]. Thus, to evaluate the production of biogenic amines by probiotic bacteria during the in vitro protein digestion, concentrations of biogenic amines were evaluated. In the present study, tyramine was not detected in any of the samples and histamine was detected only after protein digestion, where all treatments had similar concentrations (Figure 3). For cadaverine, which is among the most common biogenic amines found in fermented foods [37], we noted significant increases with some probiotics compared to the control in pea protein digests; however, the concentrations were at a similar level as with all soy protein digests including the soy protein control (Figure 3). The highest concentration of cadaverine detected in probiotic and control digests would account to approximately 120 mg cadaverine per 1 kg pea protein. For comparison, cadaverine can be found at much higher concentrations in fermented foods such as cheese, up to 3170 mg per kg product [37]. It is thus unlikely that the tested probiotic strains produce biogenic amines at harmful levels under digestive conditions.

Despite being a very useful tool for screening purposes with controlled and equal test conditions for each of the probiotic strains, the applied in vitro digestion model has some limitations. In general, the most critical limitation is the lack of interaction between the host, food, and the gastrointestinal microbiota. While there is no removal of the digestive products via absorption or transition in the in vitro model, inhibition of enzymatic activities may take place and the digestive process might not be complete. In addition, in the in vivo situation, there may be a possibility for synergistic protein digestion between a probiotic strain and the intestinal microbiota, leading to improved protein bioavailability. Due to a more homogenous environment with a fixed acidic pH in the gastric phase compared to in vivo, in vitro conditions are probably also harsher to the probiotic strains, resulting in lower survival rates with some of the strains. The static digestion model with a constant pH (~3.0) for the gastric phase may not be accurate in predicting probiotic survival [19], since the digestive conditions in vivo are more dynamic and affected, for instance, by the composition and buffering capacity of ingested food. The survival of probiotics under simulated digestive conditions has been studied by others, confirming differences in survival between bacteria species and strains [38,39,40]. In agreement with our results on the loss of CFU counts after UGIT simulation (Figure 4, Supplementary Table S5), Madureira et al. [39] demonstrated 2–5 log reductions in lactobacilli CFU counts. *L. lactis* Ll-23 was not detected in whey protein digesta samples, and in soy protein digests, *L. lactis* Ll-23 had the highest reduction in bacterial counts (Figure 4). Our result is also in line with the study by Faye et al. [40] who reported a poorer survival of *Lactococcus* strains compared to *Lactobacillus* strains. Most importantly, the survival of probiotics is largely affected by the delivery format [41]. In our study, probiotics were inoculated as live cultures and higher probiotic counts were observed in soy and pea protein digests. This is likely due to the lower solubility of soy and pea protein in the digestive fluids compared to whey protein, thus providing better matrix/protection for the bacteria against the digestive conditions such as low pH.

The advantage of using a harmonized digestion protocol is to be able to conduct reproducible studies and compare data between research groups. Despite some differences related to the composition of protein substrates and protein boluses, our results on free amino acid profiles and concentrations of free amino acids from the digestion of soy, pea, and whey protein without probiotics (Supplementary Figure S1) are very similar to results reported by Santos-Hernandez et al. [42]. In all soy and pea protein intestinal digests, the most abundant free EAAs were leucine, lysine, and phenylalanine and the most abundant free non-essential amino acid was arginine, followed by tyrosine (Figure 2A,B). This result is in line with study by Santos-Hernandez et al. [42] who also used the INFOGEST protocol on the static digestion model [17] in their study on the digestibility of plant protein. In addition, the DH value for intestinal digests of whey protein (34%; Table 2) was at the same level as reported by Ariens et al. [43] using a modified INFOGEST protocol. For soy and pea protein, similar results on DH values have been reported by others using a static digestion model [42,44,45]. In protein bioaccessibility studies, DH values and the release of free amino acids are often lower in static digestion models due to enzyme inhibition of the protein hydrolysis end-products [46]. Taken together, our results show good reproducibility of the simulated digestion model when comparing results reported by others on soy and pea protein digestion and probiotic survival. 

Overall, our results demonstrate that specific probiotic LAB can increase the digestion of soy and pea protein and improve the bioaccessibility of amino acids in the simulated digestive environment of the upper GI tract. Based on the measured parameters, *L. acidophilus* NCFM was most prominently effective with soy and pea protein digestion, and both *B. lactis* strains B420 and Bl-04 demonstrated a good capacity to increase the bioaccessibility of the tested plant protein ingredients. The results should not be generalized to all plant proteins and protein sources but should be individually evaluated. In addition, mechanistic studies are needed to provide a deeper understanding on the interaction between different protein sources and probiotics under digestive conditions. To translate our in vitro results to real-world nutrition, clinical trials are necessary to validate the effectiveness of the most promising probiotics. Future clinical research should address the acute effects of probiotics on protein bioaccessibility but also the potential long-term effects on protein nutrition.

## 5. Conclusions

Over the past decade, plant-based and milk alternatives have markedly increased in popularity. Approaches to increase the bioaccessibility of plant protein may help individuals better meet their daily needs of protein. Probiotics may increase the bioaccessibility and bioavailability of amino acids by aiding protein digestion in the gastrointestinal tract. Different probiotic strains appear to act differently depending on the protein source, which is likely affected by their enzymatic systems and metabolic pathways, and where strain survival in the digestive tract has an impact, too. Although our results from the in vitro digestion model provide an interesting strategy to improve plant protein digestion and the bioaccessibility of amino acids, human clinical research is needed to evaluate the efficacy of probiotics on amino acid absorption and bioavailability in vivo. Moreover, investigating the effect of probiotics on the digestion of other plant protein sources and protein matrices may be of future interest together with more in-depth mechanistical work.

## Figures and Tables

**Table 1:** Probiotic strains and growth media.

Species	Strain	Media
*Bifidobacterium animalis* subsp. *lactis*	B420	*Bifidobacterium* medium 58
*Bifidobacterium animalis* subsp. *lactis*	Bl-04	*Bifidobacterium* medium 58
*Lactobacillus acidophilus*	NCFM	MRS
*Lacticaseibacillus rhamnosus*	HN001	MRS
*Lacticaseibacillus paracasei* subsp. *paracasei*	Lpc-37	MRS
*Lactiplantibacillus plantarum*	Lp-115	MRS
*Lactococcus lactis* subsp. *lactis*	Ll-23	MRS

**Table 2:** Free α-amino nitrogen (FAN) measured at baseline and after the in vitro digestion of soy, pea, and whey protein. Results are presented as mean (standard deviation, SD).

Treatment	FAN, Baseline (mg/g Protein)	FAN, after Digestion (mg/g Protein)	DH (%)
Soy protein			
Control	6.52 (0.30)	23.4 (3.0) ^†^	25.4 (3.2)
B420	7.31 (0.53)	27.8 (0.84) ^†^*	30.2 (0.9)
Bl-04	7.82 (0.17)	31.1 (2.5) ^†^***	33.8 (2.7) **
NCFM	8.34 (0.43)	30.8 (1.4) ^†^***	33.4 (1.5) *
HN001	7.31 (0.44)	30.6 (2.7) ^†^***	33.2 (2.9) *
Lpc-37	7.12 (0.31)	28.1 (2.7) ^†^**	30.5 (2.9)
Lp-115	7.31 (0.18)	28.7 (3.4) ^†^**	31.1 (3.7)
Ll-23	6.72 (0.16)	25.4 (2.7) ^†^	27.5 (2.9)
Pea protein			
Control	3.23 (0.29)	26.1 (2.75) ^†^	28.4 (3.0)
B420	4.21 (0.24)	29.4 (2.02) ^†^*	32.0 (2.2)
Bl-04	4.10 (0.68)	27.8 (1.68) ^†^	30.2 (1.8)
NCFM	3.77 (0.66)	30.2 (1.01) ^†^**	32.8 (1.1)
HN001	3.54 (1.16)	26.2 (3.03) ^†^	28.5 (3.3)
Lpc-37	4.21 (0.66)	24.9 (0.73) ^†^	27.0 (0.8)
Lp-115	3.13 (0.49)	27.0 (1.43) ^†^	29.3 (1.6)
Ll-23	3.31 (0.57)	25.7 (0.86) ^†^	28.0 (0.9)
Whey protein			
Control	9.6 (0.26)	37.6 (5.43) ^†^	33.6 (4.8)
B420	12.6 (1.50)	42.1 (1.64) ^†^	37.6 (1.5)
Bl-04	13.5 (0.73)	41.2 (3.66) ^†^	36.8 (3.2)
NCFM	12.1 (1.84)	35.2 (2.75) ^†^	31.4 (2.5)
HN001	14.4 (3.84)	35.0 (2.14) ^†^	31.3 (1.9)
Lpc-37	13.5 (1.84)	45.1 (2.79) ^†^*	40.2 (2.5)
Lp-115	13.1 (1.78)	38.6 (4.52) ^†^	34.5 (4.0)
Ll-23	12.1 (1.44)	39.0 (4.25) ^†^	34.8 (3.8)

^†^ Significantly different compared to the corresponding baseline. * *p* < 0.05, ** *p* < 0.01, *** *p* < 0.001, significantly different from the control

**Table 3:** Summary of results by probiotic strains.

		Soy Protein			Pea Protein		
Probiotic	Survival ^a^	Protein Solubility ^b^	Protein Hydrolysis (FAN) ^b^	Free Amino Acids ^b^	Protein Solubility ^b^	Protein Hydrolysis (FAN) ^b^	Free Amino Acids ^b^
*B. animalis* subsp. *lactis* B420	↔	↑↑	↑	↑↑	↔	↑	
*B. animalis* subsp. *lactis* Bl-04	↔	↔	↑↑	↑	↑↑	↔	Gln↑
*L. acidophilus* NCFM	↓	↔	↑↑	↑↑	↑↑	↑↑	
*L. rhamnosus* HN001	↓	↔	↑↑	↑↑	↔	↔	Asp↑↑, Met↑
*L. paracasei* subsp. paracasei Lpc-37	↓	↔	↑↑	↑ ^c^	↔	↔	Asp↑↑, Cys↑, Met↑↑
*L. plantarum* Lp-115	↓	↔	↑↑	↔	↑	↔	
*L. lactis* subsp. *lactis* Ll-23	↓	↑↑	↔	↑	↑↑	↔	

^a^ in survival: ↔ = reduction in cell count less than 0.5 log in soy and pea digests; ↓ = reduction in cell count more than 2.5 log. ^b^ in protein solubility, protein hydrolysis and total free amino acids: ↔ = not different from control after digestion; ↑ = significantly higher result compared to control after digestion, *p* < 0.05; ↑↑ = significantly higher result compared to control after digestion, *p* < 0.01. Gln = glutamine; Met = methionine; Asp = aspartic acid. ^c^ significantly higher result in free essential amino acids, *p* < 0.05, but not in total free amino acids.

## Data Availability

All data generated or analyzed during this study are included in this published article and in its supplementary information file.

## Supplementary Materials

The following supporting information can be downloaded at https://www.mdpi.com/article/10.3390/nu15183905/s1, Table S1: Soluble protein measured at the beginning and after the in vitro digestion of soy, pea, and whey protein ingredients; Table S2: The absolute values of free amino acids at the beginning (baseline) and after the simulated digestion of soy and pea protein by treatment; Table S3: Comparison of probiotic soy protein digests to control digests in terms of absolute change in free amino acid content in the soluble phase from baseline to after digestion; Table S4: Comparison of probiotic pea protein digests to control digests in terms of absolute change in free amino acid content from baseline to after digestion; Table S5: Probiotic counts as log10 colony-forming units (CFU) at baseline and after in vitro digestion of soy, pea, and whey protein. Figure S1: Profiles of free amino acids (AAs) released from whey, soy, and pea protein after the in vitro digestion; Figure S2: Concentration of biogenic amines (mg/g protein) in samples collected at the beginning and after the in vitro digestion of soy, pea, and whey protein.
