# Supplementary material for: Enhancing Bioaccessibility of Plant Protein Using Probiotics: An In Vitro Study
Source: Nutrients. 2023 Sep 7;15(18):3905. doi: 10.3390/nu15183905 (PMC10535511; doi:10.3390/nu15183905)
Supplement: Supplementary file 1 [file nutrients-15-03905-s001.zip › nutrients-2580155-supplementary.pdf]

Supplementary Material

Enhancing bioaccessibility of plant protein by probiotics: an *in vitro* study

Journal:

Nutrients

Authors:

Maija Marttinen[1]*, Mehreen Anjum[1], Markku T. Saarinen[1], Ilmari Ahonen[2], Markus J. Lehtinen[1], Päivi Nurminen[1], Arja Laitila[1]

Affiliations:

[1] IFF Health & Bioscience, Danisco Sweeteners Oy, Sokeritehtaantie 20, 02460 Kantvik, Finland;

[2] Vincit Plc, Helsinginkatu 15, 20500 Turku, Finland

*Corresponding author: maija.marttinen@iff.com

Supplementary table 1. Soluble protein measured at the beginning and after the *in vitro* digestion of soy, pea and whey protein ingredients. Values are mean (standard deviation, SD).

Treatment	Baseline (mg)	After digestion (mg)	Change, Δ (mg)	After digestion, relative to control
		Soy protein		
Control	430.9 (25.5)	428.5 (27.1)	-2.4 (3.6)	1.00 (0.06)
B420	395.0 (25.2)	714.4 (20.5) †****	+319.5 (40.4) ****	1.67 (0.05) ****
Bl-04	376.1 (53.9)	421.0 (65.2)	+44.9 (22.2)	0.98 (0.15)
NCFM	430.6 (62.4)	473.1 (87.7)	+42.5 (27.7)	1.10 (0.20)
HN001	428.9 (40.5)	448.2 (61.9)	+19.3 (77.2)	1.05 (0.14)
Lpc-37	431.5 (52.7)	449.1 (58.6)	+17.6 (28.4)	1.05 (0.14)
Lp-115	434.8 (81.2)	453.4 (66.7)	+18.5 (18.2)	1.06 (0.16)
Ll-23	522.0 (74.8)	649.4 (90.6) †***	+127.4 (54.7) **	1.52 (0.21) **
		Pea protein		
Control	96.8 (17.8)	319.3 (27.3) †	+222.6 (29.3)	1.00 (0.09)
B420	85.5 (9.3)	312.4 (44.6) †	+226.9 (52.0)	0.98 (0.14)
Bl-04	109.5 (15.4)	429.9 (55.5) †****	+320.5 (48.2) *	1.35 (0.17) **
NCFM	101.3 (25.3)	419.1 (14.6) †***	+317.8 (26.8) [a]	1.31 (0.05) **
HN001	85.6 (18.0)	322.7 (16.9) †	+237.1 (33.5)	1.01 (0.05)
Lpc-37	94.1 (24.5)	355.9 (43.6) †	+261.7 (65.8)	1.11 (0.14)
Lp-115	87.4 (23.9)	395.8 (9.1) †**	+308.4 (31.8) [a]	1.24 (0.03) *
Ll-23	107.1 (7.0)	473.9 (19.0) †****	+366.8 (25.7) **	1.48 (0.06) ****
		Whey protein		
Control	937.9 (70.3)	933.8 (96.7)	-4.1 (26.7)	1.00 (0.10)
B420	974.5 (74.0)	1052 (60.4)	+77.2 (33.0)	1.13 (0.06)
Bl-04	848.7 (9.1)	924.6 (106.5)	+75.9 (111.7)	0.99 (0.11)
NCFM	959.7 (124.5)	941.1 (111.9)	-18.6 (15.2)	1.01 (0.12)
HN001	959.0 (117.6)	948.7 (147.2)	-10.3 (49.9)	1.02 (0.16)
Lpc-37	1056.3 (138.5)	1076.1 (112.5)	+19.9 (33.8)	1.15 (0.12)
Lp-115	978.2 (43.7)	960.6 (77.1)	-17.5 (41.3)	1.03 (0.08)
Ll-23	878.4 (91.1)	873.5 (84.6)	-4.9 (12.9)	0.94 (0.09)

† Significantly different from the baseline;
* $p<0.05$, **$p<0.01$, ***$p<0.001$, ****$p<0.0001$, significantly different from the control;
[a] $0.05 \le p < 0.1$, a tendency towards a difference from the control.

B420 = *Bifidobacterium animalis* subsp. *lactis* B420; Bl-04 = *B. animalis* subsp. *lactis* Bl-04; NCFM = *Lactobacillus acidophilus* NCFM; HN001 = *Lacticaseibacillus rhamnosus* HN001; Lpc-37 = *Lacticaseibacillus paracasei* subsp. *paracasei* Lpc-37; Lp-115 = *Lactiplantibacillus plantarum* Lp-115; and Ll-23 = *Lactococcus lactis* subsp. *lactis* Ll-23.

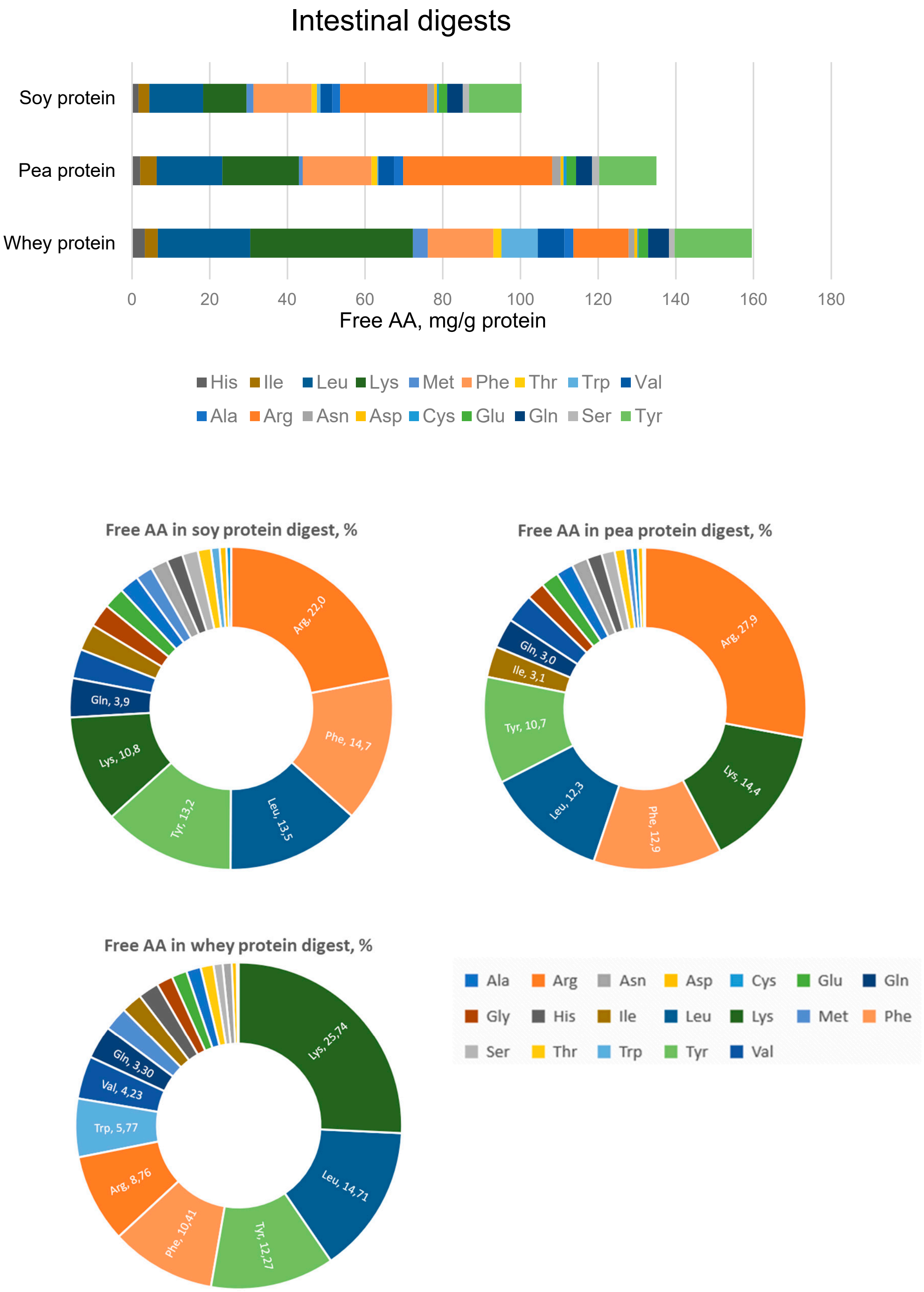

Supplementary figure 1. Profiles of free amino acids (AA) released from whey, soy and pea protein after the *in vitro* digestion. Essential amino acids (EAA): histidine (His), isoleucine (Ile), leucine (Leu), lysine (Lys), methionine (Met), phenylalanine (Phe), threonine (Thr) and valine (Val)

Supplementary table 2. The absolute values of free amino acids at the beginning (baseline) and after the simulated digestion of soy and pea protein by treatment. The table shows mean (mg/g protein) followed by standard deviation in brackets.

		Soy protein		Pea protein	
**Amino acid**	Treatment	Baseline (mg/g protein)	After digestion (mg/g protein)	Baseline (mg/g protein)	After digestion (mg/g protein)
**Alanine**	Control	0.29 (0.09)	2.03 (0.70) ***	0.33 (0.02)	2.38 (0.15) ***
	B420	0.35 (0.00)	3.13 (0.21) ***	0.35 (0.01)	2.63 (0.02) ***
	Bl-04	0.47 (0.19)	2.82 (0.37) ***	0.36 (0.01)	2.83 (0.27) ***
	NCFM	0.47 (0.11)	3.53 (0.27) ***	0.40 (0.07)	2.60 (0.27) ***
	HN001	0.43 (0.14)	3.38 (0.55) ***	0.45 (0.10)	2.65 (0.30) ***
	Lpc-37	0.41 (0.10)	2.92 (0.43) ***	0.49 (0.02)	2.80 (0.21) ***
	Lp-115	0.33 (0.08)	2.24 (0.74) ***	0.38 (0.01)	2.45 (0.06) ***
	Ll-23	0.35 (0.02)	2.88 (0.21) ***	0.42 (0.07)	2.73 (0.24) ***
**Arginine**	Control	3.46 (0.56)	22.45 (5.96) ***	2.10 (0.36)	38.36 (1.14) ***
	B420	3.79 (0.03)	27.64 (2.99) ***	2.52 (0.06)	39.96 (0.16) ***
	Bl-04	4.36 (0.89)	31.16 (8.01) ***	2.28 (0.47)	34.88 (10.55) ***
	NCFM	4.47 (0.53)	27.23 (4.38) ***	2.94 (0.49)	39.50 (2.21) ***
	HN001	4.34 (0.66)	27.00 (6.06) ***	3.16 (0.88)	39.83 (2.64) ***
	Lpc-37	4.06 (0.44)	24.43 (2.25) ***	3.47 (0.53)	40.79 (2.18) ***
	Lp-115	3.60 (0.40)	24.66 (3.25) ***	2.66 (0.29)	40.27 (2.26) ***
	Ll-23	3.98 (0.24)	26.87 (1.86) ***	2.90 (0.44)	41.76 (0.77) ***
**Asparagine**	Control	0.31 (0.06)	1.75 (0.41) ***	0.35 (0.01)	2.19 (0.09) ***
	B420	0.23 (0.02)	2.33 (0.30) ***	0.28 (0.02)	2.16 (0.21) ***
	Bl-04	0.37 (0.16)	2.20 (0.27) ***	0.31 (0.03)	2.33 (0.15) ***
	NCFM	0.42 (0.09)	2.92 (0.35) ***	0.39 (0.10)	2.36 (0.20) ***
	HN001	0.38 (0.10)	2.76 (0.62) ***	0.48 (0.11)	2.47 (0.34) ***
	Lpc-37	0.37 (0.08)	2.28 (0.38) ***	0.53 (0.04)	2.61 (0.20) ***
	Lp-115	0.35 (0.05)	1.93 (0.43) ***	0.37 (0.00)	2.23 (0.06) ***
	Ll-23	0.30 (0.07)	2.15 (0.15) ***	0.44 (0.09)	2.52 (0.27) ***
**Aspartic acid**	Control	0.14 (0.05)	0.68 (0.02) ***	0.14 (0.01)	0.71 (0.01) ***
	B420	0.16 (0.00)	0.94 (0.05) **	0.15 (0.01)	0.74 (0.02) ***
	Bl-04	0.19 (0.04)	0.94 (0.03) ***	0.17 (0.04)	0.81 (0.15) ***

		Soy protein		Pea protein	
**Amino acid**	Treatment	Baseline (mg/g protein)	After digestion (mg/g protein)	Baseline (mg/g protein)	After digestion (mg/g protein)
	NCFM	0.26 (0.06)	1.21 (0.09)	0.19 (0.08)	0.88 (0.28) ***
	HN001	0.24 (0.04)	1.12 (0.12)	0.22 (0.09)	1.01 (0.35)
	Lpc-37	0.24 (0.05)	1.00 (0.23) ***	0.27 (0.00)	1.22 (0.04)
	Lp-115	0.24 (0.05)	1.02 (0.06) **	0.18 (0.05)	0.64 (0.30) ***
	Ll-23	0.15 (0.02)	0.71 (0.19) ***	0.22 (0.07)	0.89 (0.47) ***
**Cystine**	Control	0.08 (0.00)	0.51 (0.18) ***	0.08 (0.00)	0.80 (0.04) ***
	B420	0.08 (0.00)	0.65 (0.03) ***	0.09 (0.00)	0.64 (0.05) ***
	Bl-04	0.13 (0.08)	0.80 (0.25) ***	0.13 (0.08)	0.74 (0.18) ***
	NCFM	0.08 (0.00)	0.89 (0.09) *	0.16 (0.07)	0.83 (0.22) ***
	HN001	0.08 (0.00)	0.89 (0.08) *	0.18 (0.09)	1.02 (0.23) *
	Lpc-37	0.12 (0.08)	0.84 (0.21) ***	0.24 (0.00)	1.17 (0.04)
	Lp-115	0.12 (0.08)	0.52 (0.36) ***	0.17 (0.05)	0.89 (0.24) ***
	Ll-23	0.08 (0.00)	0.62 (0.03) ***	0.17 (0.08)	0.96 (0.24) ***
**Glutamic acid**	Control	0.40 (0.13)	2.16 (0.27) ***	0.47 (0.01)	2.46 (0.04) ***
	B420	0.27 (0.00)	2.24 (0.14) ***	0.28 (0.02)	2.06 (0.06) ***
	Bl-04	0.35 (0.10)	2.27 (0.12) ***	0.34 (0.11)	2.14 (0.24) ***
	NCFM	0.55 (0.09)	3.00 (0.15) ***	0.45 (0.15)	2.57 (0.33) ***
	HN001	0.51 (0.08)	2.79 (0.48) ***	0.55 (0.11)	2.76 (0.46) ***
	Lpc-37	0.50 (0.09)	2.52 (0.44) ***	0.61 (0.00)	3.03 (0.09) ***
	Lp-115	0.54 (0.03)	2.42 (0.27) ***	0.39 (0.12)	1.88 (0.77) ***
	Ll-23	0.38 (0.08)	2.23 (0.03) ***	0.53 (0.09)	2.40 (1.01) ***
**Glutamine**	Control	0.63 (0.24)	4.03 (0.68) ***	0.58 (0.03)	4.13 (0.30) ***
	B420	0.82 (0.03)	5.84 (0.59) ***	0.62 (0.01)	4.77 (0.30) ***
	Bl-04	1.25 (0.72)	5.33 (0.65) ***	0.63 (0.02)	5.57 (0.91) ***
	NCFM	1.24 (0.35)	7.02 (0.75) ***	0.86 (0.25)	4.67 (0.72) ***
	HN001	1.12 (0.43)	6.69 (1.27) ***	1.09 (0.39)	4.78 (0.98) ***
	Lpc-37	1.02 (0.31)	6.07 (1.11) ***	1.25 (0.15)	5.24 (0.60) ***
	Lp-115	0.76 (0.27)	5.07 (0.76) ***	0.74 (0.03)	4.45 (0.10) ***
	Ll-23	0.85 (0.07)	5.47 (0.48) ***	0.94 (0.28)	5.14 (0.68) ***

		Soy protein		Pea protein	
Amino acid	Treatment	Baseline (mg/g protein)	After digestion (mg/g protein)	Baseline (mg/g protein)	After digestion (mg/g protein)
**Glycine**	Control	0.16 (0.01)	2.39 (0.23) ***	0.16 (0.00)	2.50 (0.07) ***
	B420	0.10 (0.00)	2.52 (0.15) ***	0.10 (0.00)	2.75 (0.18) ***
	Bl-04	0.13 (0.03)	2.43 (0.30) ***	0.12 (0.03)	2.45 (0.30) ***
	NCFM	0.18 (0.06)	2.77 (0.02) ***	0.16 (0.05)	2.71 (0.16) ***
	HN001	0.17 (0.05)	2.77 (0.22) ***	0.19 (0.04)	2.76 (0.13) ***
	Lpc-37	0.17 (0.05)	2.39 (0.39) ***	0.21 (0.01)	2.85 (0.02) ***
	Lp-115	0.19 (0.01)	2.27 (0.35) ***	0.15 (0.02)	2.51 (0.04) ***
	Ll-23	0.13 (0.02)	2.53 (0.11) ***	0.17 (0.05)	2.73 (0.21) ***
**Histidine**	Control	0.33 (0.03)	1.66 (0.27) ***	0.38 (0.01)	2.09 (0.08) ***
	B420	0.24 (0.04)	2.28 (0.44) ***	0.30 (0.05)	2.10 (0.42) ***
	Bl-04	0.38 (0.18)	2.21 (0.43) ***	0.36 (0.06)	2.38 (0.14) ***
	NCFM	0.44 (0.09)	2.39 (0.16) ***	0.40 (0.13)	2.29 (0.24) ***
	HN001	0.42 (0.09)	2.33 (0.24) ***	0.51 (0.10)	2.38 (0.34) ***
	Lpc-37	0.41 (0.07)	2.17 (0.31) ***	0.55 (0.03)	2.50 (0.15) ***
	Lp-115	0.41 (0.02)	2.01 (0.29) ***	0.42 (0.04)	2.25 (0.10) ***
	Ll-23	0.30 (0.09)	1.94 (0.15) ***	0.46 (0.09)	2.41 (0.24) ***
**Isoleucine**	Control	0.24 (0.08)	2.79 (1.24) ***	0.32 (0.02)	4.26 (0.36) ***
	B420	0.31 (0.00)	5.09 (0.32) ***	0.38 (0.01)	4.75 (0.16) ***
	Bl-04	0.47 (0.25)	4.76 (0.33) ***	0.38 (0.02)	5.07 (0.26) ***
	NCFM	0.47 (0.13)	5.52 (0.43) ***	0.44 (0.08)	4.58 (0.58) ***
	HN001	0.44 (0.17)	5.23 (0.94) ***	0.51 (0.15)	4.73 (0.77) ***
	Lpc-37	0.39 (0.10)	4.65 (0.77) ***	0.56 (0.07)	4.99 (0.54) ***
	Lp-115	0.31 (0.06)	3.46 (1.54) ***	0.40 (0.02)	4.53 (0.23) ***
	Ll-23	0.31 (0.01)	4.68 (0.34) ***	0.44 (0.08)	4.99 (0.34) ***
**Leucine**	Control	1.98 (0.86)	13.84 (2.32) ***	1.10 (0.09)	16.90 (1.04) ***
	B420	2.48 (0.04)	19.64 (1.10) ***	1.28 (0.02)	16.82 (0.31) ***
	Bl-04	3.08 (1.09)	18.37 (1.24) ***	1.22 (0.18)	18.30 (1.42) ***
	NCFM	3.29 (0.52)	20.94 (1.27) ***	1.47 (0.22)	17.23 (0.86) ***
	HN001	3.10 (0.79)	20.30 (3.07) ***	1.66 (0.47)	18.04 (1.95) ***

		Soy protein		Pea protein	
**Amino acid**	Treatment	Baseline (mg/g protein)	After digestion (mg/g protein)	Baseline (mg/g protein)	After digestion (mg/g protein)
	Lpc-37	2.82 (0.52)	18.41 (2.01) ***	1.79 (0.29)	18.54 (1.56) ***
	Lp-115	2.04 (0.78)	15.68 (3.39) ***	1.33 (0.03)	17.80 (1.13) ***
	Ll-23	2.64 (0.22)	18.98 (1.27) ***	1.47 (0.21)	19.12 (0.65) ***
**Lysine**	Control	1.67 (0.54)	11.21 (2.76) ***	0.80 (0.10)	19.74 (0.91) ***
	B420	1.95 (0.07)	14.80 (2.15) ***	0.82 (0.03)	19.98 (0.37) ***
	Bl-04	2.28 (0.50)	16.29 (3.60) ***	0.81 (0.06)	17.60 (4.59) ***
	NCFM	2.32 (0.26)	15.08 (1.95) ***	1.08 (0.20)	20.18 (1.05) ***
	HN001	2.23 (0.34)	15.04 (3.36) ***	1.22 (0.34)	20.65 (1.53) ***
	Lpc-37	2.12 (0.22)	13.57 (1.51) ***	1.36 (0.15)	21.29 (1.26) ***
	Lp-115	1.75 (0.47)	12.35 (2.02) ***	0.97 (0.11)	20.40 (1.10) ***
	Ll-23	2.06 (0.15)	14.64 (1.41) ***	1.09 (0.22)	21.47 (0.83) ***
**Methionine**	Control	0.29 (0.09)	1.75 (0.23) ***	0.09 (0.00)	0.94 (0.03) **
	B420	0.32 (0.00)	2.43 (0.15) ***	0.09 (0.00)	0.94 (0.04) **
	Bl-04	0.44 (0.13)	1.96 (0.61) ***	0.13 (0.06)	1.51 (0.93)
	NCFM	0.48 (0.10)	2.64 (0.20) ***	0.13 (0.07)	1.09 (0.17)
	HN001	0.45 (0.11)	2.59 (0.33) ***	0.16 (0.07)	1.20 (0.27)
	Lpc-37	0.43 (0.08)	2.37 (0.31) ***	0.20 (0.00)	1.32 (0.08) *
	Lp-115	0.36 (0.09)	2.19 (0.28) ***	0.12 (0.06)	1.09 (0.12)
	Ll-23	0.36 (0.04)	2.28 (0.12) ***	0.16 (0.06)	1.17 (0.20)
**Phenylalanine**	Control	2.12 (0.59)	14.97 (1.28) ***	1.02 (0.07)	17.74 (0.61) ***
	B420	2.49 (0.03)	19.25 (0.79) ***	1.14 (0.02)	17.60 (0.21) ***
	Bl-04	3.12 (1.17)	18.40 (0.82) ***	1.10 (0.13)	18.57 (0.77) ***
	NCFM	3.00 (0.34)	19.51 (1.04) ***	1.30 (0.16)	17.66 (0.75) ***
	HN001	2.90 (0.57)	19.18 (2.34) ***	1.44 (0.36)	18.37 (1.54) ***
	Lpc-37	2.63 (0.33)	17.76 (1.43) ***	1.54 (0.23)	18.72 (1.39) ***
	Lp-115	2.16 (0.47)	16.17 (1.96) ***	1.23 (0.02)	18.59 (1.24) ***
	Ll-23	2.62 (0.18)	18.76 (1.13) ***	1.30 (0.14)	19.38 (0.48) ***
**Serine**	Control	0.29 (0.07)	1.60 (0.33) ***	0.28 (0.01)	1.81 (0.08) ***
	B420	0.24 (0.00)	2.03 (0.17) ***	0.20 (0.01)	1.71 (0.06) ***

		Soy protein		Pea protein	
**Amino acid**	Treatment	Baseline (mg/g protein)	After digestion (mg/g protein)	Baseline (mg/g protein)	After digestion (mg/g protein)
	Bl-04	0.34 (0.10)	1.88 (0.15) ***	0.23 (0.04)	1.83 (0.20) ***
	NCFM	0.39 (0.09)	2.45 (0.23) ***	0.28 (0.07)	1.86 (0.14) ***
	HN001	0.36 (0.10)	2.38 (0.38) ***	0.34 (0.08)	1.98 (0.22) ***
	Lpc-37	0.35 (0.08)	1.99 (0.30) ***	0.37 (0.01)	2.10 (0.09) ***
	Lp-115	0.32 (0.06)	1.72 (0.35) ***	0.27 (0.02)	1.71 (0.05) ***
	Ll-23	0.29 (0.04)	1.89 (0.09) ***	0.31 (0.06)	1.98 (0.26) ***
**Threonine**	Control	0.24 (0.07)	1.37 (0.32) *	0.23 (0.01)	1.45 (0.06) ***
	B420	0.22 (0.01)	2.02 (0.22) ***	0.17 (0.00)	1.48 (0.08) ***
	Bl-04	0.33 (0.10)	1.79 (0.21) ***	0.22 (0.07)	1.74 (0.35) ***
	NCFM	0.35 (0.07)	2.35 (0.22) ***	0.24 (0.06)	1.57 (0.16) ***
	HN001	0.35 (0.07)	2.27 (0.34) ***	0.29 (0.05)	1.69 (0.28) ***
	Lpc-37	0.34 (0.05)	1.93 (0.36) ***	0.32 (0.01)	1.78 (0.15) ***
	Lp-115	0.31 (0.06)	1.64 (0.32) ***	0.26 (0.04)	1.51 (0.12) ***
	Ll-23	0.26 (0.04)	1.82 (0.12) ***	0.27 (0.05)	1.65 (0.25) ***
**Tryptophan**	Control	0.08 (0.00)	0.90 (0.55)	0.08 (0.00)	0.30 (0.00) ***
	B420	0.23 (0.13)	2.46 (1.87) ***	0.11 (0.03)	0.82 (0.45)
	Bl-04	0.32 (0.26)	1.63 (1.66) *	0.16 (0.05)	1.65 (0.95)
	NCFM	0.22 (0.13)	1.92 (0.98) ***	0.08 (0.00)	0.56 (0.45)***
	HN001	0.28 (0.06)	2.27 (0.65) ***	0.11 (0.06)	0.44 (0.24) ***
	Lpc-37	0.27 (0.07)	2.05 (0.66) ***	0.11 (0.06)	0.44 (0.24) ***
	Lp-115	0.23 (0.01)	1.44 (0.44) **	0.13 (0.09)	0.80 (0.50) ***
	Ll-23	0.11 (0.06)	1.14 (0.73) ***	0.09 (0.02)	0.49 (0.23) ***
**Tyrosine**	Control	1.80 (0.39)	13.53 (1.87) ***	1.06 (0.08)	14.75 (0.57) ***
	B420	2.09 (0.04)	18.45 (0.80) ***	1.16 (0.01)	15.37 (0.17) ***
	Bl-04	2.67 (1.08)	16.84 (1.53) ***	1.15 (0.09)	16.73 (1.35) ***
	NCFM	2.48 (0.26)	18.43 (1.15) ***	1.29 (0.14)	14.99 (0.91) ***
	HN001	2.41 (0.44)	18.09 (2.35) ***	1.39 (0.27)	15.34 (1.26) ***
	Lpc-37	2.19 (0.26)	16.70 (1.34) ***	1.48 (0.17)	15.55 (1.15) ***
	Lp-115	1.82 (0.28)	14.46 (2.33) ***	1.27 (0.02)	15.43 (0.92) ***

		Soy protein		Pea protein	
**Amino acid**	Treatment	Baseline (mg/g protein)	After digestion (mg/g protein)	Baseline (mg/g protein)	After digestion (mg/g protein)
	Ll-23	2.19 (0.15)	17.75 (1.00) ***	1.30 (0.10)	16.05 (0.42) ***
**Valine**	Control	0.35 (0.11)	3.06 (1.31) ***	0.33 (0.01)	4.01 (0.31) ***
	B420	0.43 (0.01)	5.54 (0.36) ***	0.37 (0.01)	4.53 (0.17) ***
	Bl-04	0.64 (0.33)	4.99 (0.52) ***	0.39 (0.01)	5.09 (0.56) ***
	NCFM	0.68 (0.19)	5.93 (0.41) ***	0.48 (0.13)	4.48 (0.51) ***
	HN001	0.61 (0.24)	5.65 (0.97) ***	0.59 (0.20)	4.57 (0.67) ***
	Lpc-37	0.56 (0.16)	5.14 (0.87) ***	0.67 (0.08)	4.79 (0.43) ***
	Lp-115	0.42 (0.10)	3.78 (1.67) ***	0.43 (0.03)	4.26 (0.12) ***
	Ll-23	0.44 (0.03)	5.05 (0.36) ***	0.51 (0.14)	4.73 (0.43) ***
**Total EAA**	Control	7.30 (2.36)	51.56 (9.33) ***	4.35 (0.30)	67.43 (3.24) ***
	B420	8.66 (0.33)	73.52 (7.29) ***	4.66 (0.06)	69.02 (1.57) ***
	Bl-04	11.07 (3.93)	70.40 (6.97) ***	4.76 (0.16)	71.91 (2.48) ***
	NCFM	11.25 (1.59)	76.27 (5.55) ***	5.61 (0.91)	69.63 (4.50) ***
	HN001	10.79 (2.31)	74.86 (10.84) ***	6.49 (1.77)	72.06 (7.53) ***
	Lpc-37	9.97 (1.42)	68.05 (5.03) ***	7.11 (0.91)	74.38 (5.75) ***
	Lp-115	7.99 (2.06)	58.71 (10.86) ***	5.29 (0.24)	71.23 (2.87) ***
	Ll-23	9.11 (0.76)	69.28 (4.77) ***	5.79 (0.95)	75.41 (2.57) ***
**Total BCAA**	Control	2.57 (1.05)	19.69 (4.83) ***	1.76 (0.12)	25.17 (1.69) ***
	B420	3.22 (0.06)	30.28 (1.77) ***	2.03 (0.04)	26.09 (0.61) ***
	Bl-04	4.20 (1.67)	28.12 (2.04) ***	1.99 (0.19)	28.45 (2.17) ***
	NCFM	4.44 (0.84)	32.40 (2.10) ***	2.39 (0.42)	26.29 (1.91) ***
	HN001	4.15 (1.20)	31.18 (4.98) ***	2.76 (0.81)	27.34 (3.39) ***
	Lpc-37	3.77 (0.77)	28.20 (3.64) ***	3.03 (0.44)	28.32 (2.54) ***
	Lp-115	2.78 (0.94)	22.92 (6.59) ***	2.16 (0.01)	26.59 (1.45) ***
	Ll-23	3.39 (0.26)	28.71 (1.91) ***	2.42 (0.43)	28.84 (1.35) ***
**Total AA**	Control	14.85 (3.95)	102.70 (14.63) ***	9.90 (0.78)	137.51 (5.29) ***
	B420	16.80 (0.45)	139.28 (12.68) ***	10.40 (0.13)	141.80 (2.03) ***
	Bl-04	21.32 (6.92)	137.07 (13.25) ***	10.49 (0.41)	142.20 (9.06) ***
	NCFM	21.78 (3.18)	145.73 (12.33) ***	12.74 (2.05)	142.60 (9.44) ***

		Soy protein		Pea protein	
**Amino acid**	Treatment	Baseline (mg/g protein)	After digestion (mg/g protein)	Baseline (mg/g protein)	After digestion (mg/g protein)
	HN001	20.82 (4.34)	142.74 (22.46) ***	14.55 (3.89)	146.67 (14.40) ***
	Lpc-37	19.40 (2.79)	129.19 (9.16) ***	16.04 (1.82)	151.73 (10.28) ***
	Lp-115	16.26 (3.32)	115.02 (13.97) ***	11.87 (0.58)	143.70 (5.17) ***
	Ll-23	17.79 (1.41)	132.37 (8.17) ***	13.19 (2.18)	152.57 (5.66) ***

*p<0.05, **p<0.01, and ***p<0.001, significant difference in terms of absolute change in amino acid concentration from baseline to after digestion within treatment. The data were log-transformed and analyzed using a robust linear model that is not affected by the heavy-tailed distribution of the data (outliers). p-values are corrected for false discovery rate (FDR) using Benjamini-Hochberg method.

B420 = *Bifidobacterium animalis* subsp. *lactis* B420; Bl-04 = *B. animalis* subsp. *lactis* Bl-04; NCFM = *Lactobacillus acidophilus* NCFM; HN001 = *Lacticaseibacillus rhamnosus* HN001; Lpc-37 = *Lacticaseibacillus paracasei* subsp. *paracasei* Lpc-37; Lp-115 = *Lactiplantibacillus plantarum* Lp-115; and Ll-23 = *Lactococcus lactis* subsp. *lactis* Ll-23.

Supplementary table 3. Comparison of probiotic **soy protein** digests to control digests in terms of absolute change in free amino acid content in the soluble phase from baseline to after digestion. The comparisons are reported as ratios against the control treatment where a ratio of 1.0 denotes no difference, ratio < 1 less than control and ratio > 1 higher than control. Standard error of the estimate is reported in parentheses.

Amino acid	B420	Bl-04	NCFM	HN001	Lpc-37	Lp-115	Ll-23
Alanine	1.42 (0.16) **	1.20 (0.14)	1.55 (0.18) ***	1.55 (0.18) ***	1.30 (0.15) *	1.10 (0.13)	1.29 (0.15) *
Arginine	1.13 (0.13)	1.12 (0.13)	1.09 (0.12)	1.08 (0.12)	0.96 (0.11)	1.02 (0.12)	1.08 (0.12)
Asparagine	1.37 (0.16) *	1.18 (0.14)	1.61 (0.18) ***	1.64 (0.19) ***	1.24 (0.14)	1.09 (0.12)	1.20 (0.14)
Aspartic acid	1.43 (0.16) **	1.39 (0.16) **	1.76 (0.20) ***	1.62 (0.18) ***	1.34 (0.15) *	1.44 (0.17) **	0.91 (0.10)
Cystine	1.14 (0.13)	1.23 (0.14)	1.63 (0.19) ***	1.64 (0.19) ***	1.43 (0.16) **	0.46 (0.05) ***	1.08 (0.12)
Glutamic acid	1.12 (0.13)	1.09 (0.12)	1.40 (0.16) **	1.36 (0.16) *	1.11 (0.13)	1.07 (0.12)	1.06 (0.12)
Glutamine	1.51 (0.17) **	1.23 (0.14)	1.74 (0.20) ***	1.72 (0.20) ***	1.57 (0.18) ***	1.30 (0.15) *	1.39 (0.16) **
Glycine	1.09 (0.12)	1.05 (0.12)	1.16 (0.13)	1.17 (0.13)	1.00 (0.11)	0.95 (0.11)	1.08 (0.12)
Histidine[a]	1.55 (0.18) ***	1.35 (0.15) *	1.42 (0.16) **	1.39 (0.16) **	1.30 (0.15) *	1.20 (0.14)	1.19 (0.14)
Isoleucine[a,b]	1.66 (0.19) ***	1.49 (0.17) **	1.75 (0.20) ***	1.69 (0.19) ***	1.52 (0.17) **	1.24 (0.14)	1.51 (0.17) **
Leucine[a,b]	1.46 (0.17) **	1.30 (0.15) *	1.50 (0.17) **	1.47 (0.17) **	1.33 (0.15) *	1.16 (0.13)	1.39 (0.16) **
Lysine[a]	1.40 (0.16) **	1.42 (0.16) **	1.38 (0.16) **	1.42 (0.16) **	1.22 (0.14)	1.09 (0.12)	1.33 (0.15) *
Methionine[a]	1.44 (0.16) **	1.18 (0.13)	1.48 (0.17) **	1.46 (0.17) **	1.33 (0.15) *	1.24 (0.14)	1.32 (0.15) *
Phenylalanine[a]	1.31 (0.15) *	1.19 (0.14)	1.29 (0.15) *	1.26 (0.14)	1.18 (0.13)	1.09 (0.12)	1.26 (0.14)
Serine	1.30 (0.15) *	1.13 (0.13)	1.48 (0.17) **	1.49 (0.17) **	1.19 (0.14)	1.07 (0.12)	1.17 (0.13)
Threonine[a]	1.50 (0.17) **	1.23 (0.14)	1.67 (0.19) ***	1.66 (0.19) ***	1.39 (0.16) **	1.16 (0.13)	1.31 (0.15) *
Tryptophan[a]	3.17 (0.38) ***	0.85 (0.10)	1.55 (0.18) **	2.30 (0.27) ***	2.07 (0.25) ***	1.34 (0.16) *	1.39 (0.17) *
Tyrosine	1.41 (0.16) **	1.22 (0.14)	1.37 (0.16) *	1.34 (0.15) *	1.24 (0.14)	1.11 (0.13)	1.34 (0.15) *
Valine[a,b]	1.70 (0.19) ***	1.44 (0.16) **	1.75 (0.20) ***	1.69 (0.19) ***	1.56 (0.18) ***	1.27 (0.15)	1.53 (0.17) **
total EAA[a]	1.51 (0.17) **	1.39 (0.16) **	1.52 (0.17) **	1.51 (0.17) **	1.36 (0.16) *	1.22 (0.14)	1.41 (0.16) **
total BCAA[b]	1.56 (0.18) ***	1.38 (0.16) **	1.61 (0.18) ***	1.57 (0.18) ***	1.42 (0.16) **	1.22 (0.14)	1.46 (0.17) **
Total AA	1.42 (0.16) **	1.34 (0.15) *	1.43 (0.16) **	1.43 (0.16) **	1.27 (0.15)	1.14 (0.13)	1.33 (0.15) *

[a] EAA, essential amino acid; [b] BCAA, branched chain amino acid. Statistically significant different to control digestion without probiotic; * *p*<0.05, ***p*<0.01, ****p*<0.001.

B420 = *Bifidobacterium animalis* subsp. *lactis* B420; Bl-04 = *B. animalis* subsp. *lactis* Bl-04; NCFM = *Lactobacillus acidophilus* NCFM; HN001 = *Lacticaseibacillus rhamnosus* HN001; Lpc-37 = *Lacticaseibacillus paracasei* subsp. *paracasei* Lpc-37; Lp-115 = *Lactiplantibacillus plantarum* Lp-115; and Ll-23 = *Lactococcus lactis* subsp. *lactis* Ll-23.

Supplementary table 4. Comparison of probiotic **pea protein** digests to control digests in terms of absolute change in free amino acid content from baseline to after digestion. The comparisons are reported as ratios against the control treatment where a ratio of 1.0 denotes no difference, ratio < 1 less than control and ratio > 1 higher than control. Standard error of the estimate is reported in parentheses.

Amino acid	B420	Bl-04	NCFM	HN001	Lpc-37	Lp-115	Ll-23
Alanine	1.11 (0.08)	1.18 (0.09)	1.05 (0.08)	1.07 (0.08)	1.13 (0.08)	1.01 (0.08)	1.12 (0.08)
Arginine	1.03 (0.08)	1.02 (0.08)	1.01 (0.08)	1.01 (0.08)	1.03 (0.08)	1.04 (0.08)	1.07 (0.07)
Asparagine	1.04 (0.08)	1.10 (0.08)	1.07 (0.08)	1.09 (0.08)	1.14 (0.08)	1.01 (0.08)	1.13 (0.08)
Aspartic acid	1.04 (0.08)	1.11 (0.08)	1.24 (0.09)	1.59 (0.12) ***	1.67 (0.12) ***	0.88 (0.07)	0.97 (0.07)
Cystine	0.76 (0.06) **	0.85 (0.06)	0.89 (0.07)	1.23 (0.09)	1.30 (0.10) *	0.90 (0.07)	1.08 (0.08)
Glutamic acid	0.90 (0.07)	0.92 (0.07)	1.09 (0.08)	1.14 (0.09)	1.22 (0.09)	0.90 (0.07)	1.03 (0.07)
Glutamine	1.17 (0.09)	1.36 (0.10) **	1.06 (0.08)	1.03 (0.08)	1.16 (0.09)	1.05 (0.08)	1.17 (0.08)
Glycine	1.13 (0.08)	1.03 (0.08)	1.09 (0.08)	1.10 (0.08)	1.13 (0.08)	1.01 (0.08)	1.09 (0.08)
Histidine[a]	1.14 (0.08)	1.18 (0.09)	1.11 (0.08)	1.11 (0.08)	1.14 (0.09)	1.07 (0.08)	1.14 (0.08)
Isoleucine[a,b]	1.12 (0.08)	1.20 (0.09)	1.07 (0.08)	1.08 (0.08)	1.15 (0.09)	1.06 (0.08)	1.16 (0.08)
Leucine[a,b]	0.98 (0.07)	1.07 (0.08)	1.00 (0.07)	1.04 (0.08)	1.06 (0.08)	1.04 (0.08)	1.12 (0.08)
Lysine[a]	1.01 (0.08)	0.98 (0.07)	1.01 (0.08)	1.03 (0.08)	1.05 (0.08)	1.03 (0.08)	1.08 (0.08)
Methionine[a]	1.00 (0.07)	1.08 (0.08)	1.16 (0.09)	1.26 (0.09) *	1.31 (0.10) **	1.14 (0.09)	1.19 (0.08)
Phenylalanine[a]	0.98 (0.07)	1.04 (0.08)	0.98 (0.07)	1.01 (0.08)	1.03 (0.08)	1.04 (0.08)	1.08 (0.08)
Serine	0.99 (0.07)	1.06 (0.08)	1.03 (0.08)	1.07 (0.08)	1.13 (0.08)	0.94 (0.07)	1.10 (0.08)
Threonine[a]	1.06 (0.08)	1.20 (0.09)	1.10 (0.08)	1.14 (0.09)	1.18 (0.09)	1.02 (0.08)	1.12 (0.08)
Tryptophan[a]	4.04 (0.31) ***	4.56 (0.35) ***	1.04 (0.08)	1.04 (0.08)	1.04 (0.08)	3.12 (0.24) ***	1.61 (0.12) ***
Tyrosine	1.04 (0.08)	1.13 (0.08)	1.00 (0.07)	1.02 (0.08)	1.03 (0.08)	1.03 (0.08)	1.08 (0.08)
Valine[a,b]	1.13 (0.08)	1.25 (0.09)	1.08 (0.08)	1.08 (0.08)	1.13 (0.08)	1.04 (0.08)	1.15 (0.08)
Total EAA[a]	1.02 (0.08)	1.06 (0.08)	1.01 (0.08)	1.04 (0.08)	1.07 (0.08)	1.05 (0.08)	1.10 (0.08)
Total BCAA[b]	1.03 (0.08)	1.12 (0.08)	1.02 (0.08)	1.05 (0.08)	1.09 (0.08)	1.04 (0.08)	1.13 (0.08)
Total AA	1.03 (0.08)	1.03 (0.08)	1.02 (0.08)	1.03 (0.08)	1.06 (0.08)	1.03 (0.08)	1.09 (0.08)

[a] EAA, essential amino acid; [b] BCAA, branched chain amino acid. Statistically significant different to control digestion without probiotic; **p*<0.05, ***p*<0.01, ****p*<0.001.

B420 = *Bifidobacterium animalis* subsp. *lactis* B420; Bl-04 = *B. animalis* subsp. *lactis* Bl-04; NCFM = *Lactobacillus acidophilus* NCFM; HN001 = *Lacticaseibacillus rhamnosus* HN001; Lpc-37 = *Lacticaseibacillus paracasei* subsp. *paracasei* Lpc-37; Lp-115 = *Lactiplantibacillus plantarum* Lp-115; and Ll-23 = *Lactococcus lactis* subsp. *lactis* Ll-23.

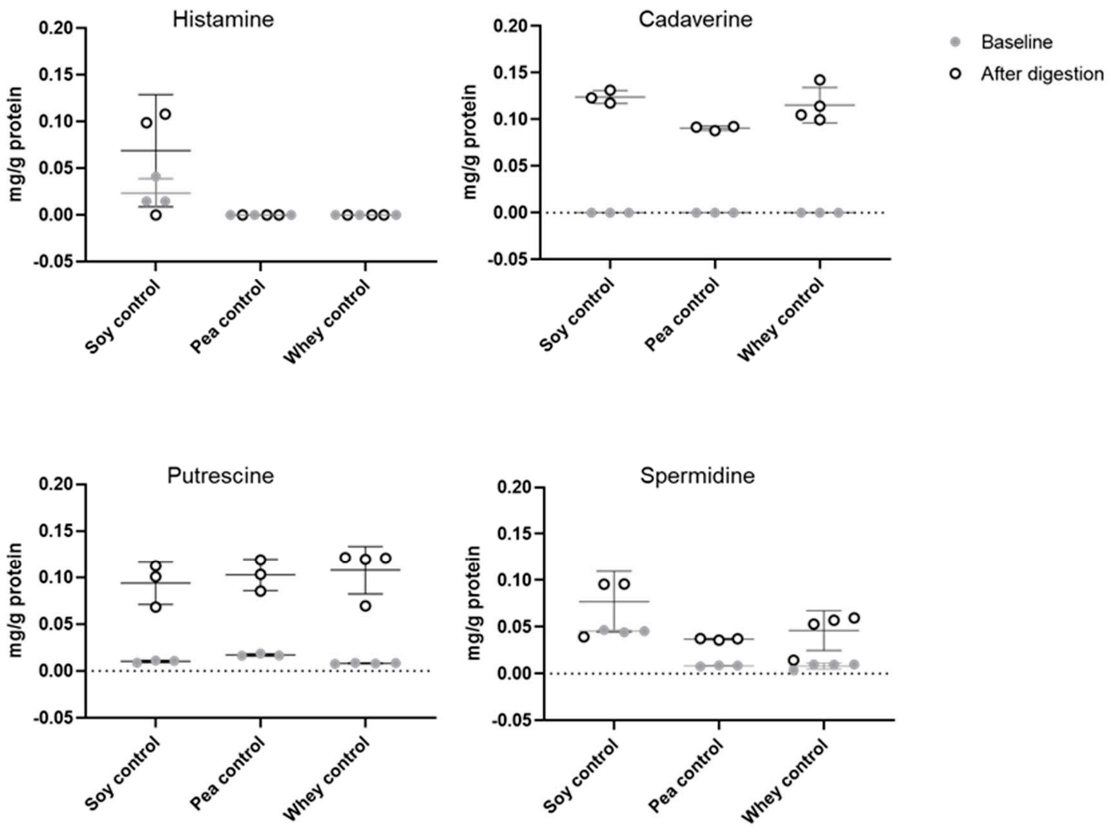

Supplementary figure 2. Concentration of biogenic amines (mg/g protein) in samples collected at the beginning and after the *in vitro* digestion of soy, pea and whey protein.

Supplementary table 5**.** Probiotic counts as log10 colony forming units (CFU) at baseline and after *in vitro* digestion of soy, pea and whey protein. Values are mean (standard deviation, SD).

Treatment	Baseline (log10 CFU)	After digestion (log10 CFU)	Change, Δ (log10 CFU)
		Soy protein	
B420	8.6 (0.19)	8.3 (0.19)	-0.30 (0.02)
Bl-04	8.9 (0.21)	8.5 (0.18)	-0.33 (0.03)
NCFM	8.6 (0.08)	5.5 (0.07)	-3.1 (0.04)
HN001	8.8 (0.08)	5.6 (0.15)	-3.2 (0.07)
Lpc-37	8.8 (0.28)	5.3 (0.16)	-3.5 (0.32)
Lp-115	9.0 (0.14)	6.4 (0.18)	-2.6 (0.16)
Ll-23	9.0 (0.06)	4.5 (0.06)	-4.5 (0.02)
		Pea protein	
B420	8.8 (0.20)	8.5 (0.14)	-0.27 (0.08)
Bl-04	8.9 (0.09)	8.7 (0.10)	-0.29 (0.03)
NCFM	8.1 (0.10)	5.4 (0.07)	-2.6 (0.08)
HN001	8.8 (0.32)	5.6 (0.11)	-3.2 (0.26)
Lpc-37	8.8 (0.11)	4.8 (0.09)	-4.0 (0.10)
Lp-115	8.8 (0.25)	6.2 (0.42)	-2.7 (0.18)
Ll-23	8.5 (0.36)	5.4 (0.27)	-3.1 (0.16)
		Whey protein	
B420	8.7 (0.23)	8.3 (0.19)	-0.35 (0.05)
Bl-04	8.6 (0.00)	7.7 (0.06)	-0.86 (0.06)
NCFM	8.4 (0.16)	4.3 (0.09)	-4.1 (0.15)
HN001	8.6 (0.01)	4.7 (0.24)	-3.9 (0.25)
Lpc-37	8.7 (0.22)	4.3 (0.17)	-4.4 (0.06)
Lp-115	8.8 (0.12)	4.6 (0.12)	-4.2 (0.11)
Ll-23	8.7 (0.09)	0.0 (0.0)	-8.7 (0.09)

B420 = *Bifidobacterium animalis* subsp. *lactis* B420; Bl-04 = *B. animalis* subsp. *lactis* Bl-04; NCFM = *Lactobacillus acidophilus* NCFM; HN001 = *Lacticaseibacillus rhamnosus* HN001; Lpc-37 = *Lacticaseibacillus paracasei* subsp. *paracasei* Lpc-37; Lp-115 = *Lactiplantibacillus plantarum* Lp-115; and Ll-23 = *Lactococcus lactis* subsp. *lactis* Ll-23.
